# Machine learning in understanding environmental variability of vibriosis in coastal waters

**DOI:** 10.1128/aem.00716-25

**Published:** 2025-08-14

**Authors:** Bailey M. Magers, Kyle D. Brumfield, Sunil Kumar, Rita R. Colwell, Antarpreet S. Jutla

**Affiliations:** 1 Geohealth and Hydrology Laboratory, Department of Environmental Engineering Sciences, University of Florida 3463, Gainesville, Florida, USA; 2 Maryland Pathogen Research Institute, University of Maryland 1068 , College Park, Maryland, USA; 3 University of Maryland Institute for Advanced Computer Studies, University of Maryland 1068 , College Park, Maryland, USA; Indiana University Bloomington, Bloomington, Indiana, USA

**Keywords:** vibriosis, *Vibrio mimicus*, *Vibrio fluvialis*, *Vibrio cholerae*, *Vibrio alginolyticus*, *Vibrio vulnificus*, *Vibrio parahaemolyticus*, *Vibrio* spp., *Vibrio*

## Abstract

*Vibrio spp.* comprise ecologically significant bacteria that thrive in warm, moderately saline water, and their incidence and proliferation are strongly influenced by environmental factors. In recent years, *Vibrio spp*. infections have been reported more frequently and over a greater geographical area along the US eastern seaboard. This study provides an analysis of *Vibrio spp*. infections, notably caused by *Vibrio alginolyticus*, *Vibrio cholerae* non-O1/non-O139, *Vibrio fluvialis*, *Vibrio mimicus*, *V. parahaemolyticus*, and *V. vulnificus*, extracted from the Centers for Disease Control and Prevention’s Cholera and Other *Vibrio* Illness Surveillance system, located within 200 km of the eastern US coast, to analyze latitudinal distribution trends between 1990 and 2019. For each *Vibrio spp*., case incidence (presence/absence) was modeled, including environmental data and employing extreme gradient boosting machine learning algorithms (XGBoost). Environmental parameters associated with the incidence of vibriosis were clustered using k-means clustering. The northern limit of total cases of vibriosis was found to have increased *ca*. 40 km/year, with *V. alginolyticus* (*ca*. 70 km/year), *V. fluvialis* (*ca*. 60 km/year), and *Vibrio parahaemolyticus* (*ca*. 60 km/year) showing the greatest latitudinal shifts. These changes were found to be linked to environmental parameters that enhance the proliferation of *Vibrio spp*. The average accuracy of the XGBoost models was 60.9%–71.0%, with temperature and salinity being the most significant predictors. Relationships among other environmental parameters were complex and nonlinear, but phytoplankton and precipitation served to differentiate the models. Clustering using k-means yielded results that supported temperature, salinity, and phytoplankton as important environmental parameters. Research in progress will aid in developing global predictive risk models for *Vibrio spp*. infections.

**IMPORTANCE:**

*Vibrio spp*. are ecologically significant bacteria, and their incidence and proliferation are strongly influenced by environmental factors. In recent years, *Vibrio spp*. infections have been reported more frequently and over a greater geographical area along the US eastern seaboard. This study provides an analysis of latitudinal distribution trends of *Vibrio spp*. infections, notably caused by *Vibrio alginolyticus*, *Vibrio cholerae* non-O1/non-O139, *Vibrio fluvialis*, *Vibrio mimicus*, *Vibrio parahaemolyticus*, and *V. vulnificus*, within 200 km of the eastern US coast. The northern limit of total cases of vibriosis was found to have increased *ca*. 40 km/year. These changes were found to be linked to environmental parameters that enhance the proliferation of *Vibrio spp*. Temperature and salinity were the most significant predictors of vibriosis case presence and absence. Phytoplankton and precipitation changes served to differentiate *Vibrio sp*. presence. Research in progress will aid in developing global predictive risk models for *Vibrio spp*. infections.

## INTRODUCTION

*Vibrio spp*. are autochthonous to riverine, estuarine, and coastal environments and also freshwater ecosystems globally (1). *Vibrio spp*. thrive in warm, moderately saline water, with incidence and proliferation heavily influenced by a variety of environmental factors (1, 2). Furthermore, *Vibrio spp*. form mutualistic associations with aquatic multicellular hosts, most notably zooplankton (3, 4), corals (5), fish (6), mollusks and bivalves (7), seagrass, sponges, and shrimps (8, 9). In addition, *Vibrio spp*. play an important role in carbon and nitrogen cycling (10, 11). However, despite their ecological significance in the aquatic ecosystem, *Vibrio spp*. cause severe infections in humans, notably *Vibrio cholerae* O1, the causative agent of pandemic cholera. Non-cholera *Vibrio spp*., notably *V. cholerae* non-O1/non-O139, *Vibrio vulnificus*, *Vibrio parahaemolyticus*, *Vibrio alginolyticus*, *Vibrio fluvialis*, and *Vibrio mimicus*, can cause acute gastroenteritis, septicemia, and other extra-intestinal infections.

In developed countries of the world, the risk of cholera has essentially been eliminated with the availability of safe water and effective sanitation. However, non-cholera *Vibrio* infections (vibriosis) persist in essentially all countries, notably associated with consumption of raw and undercooked seafood or contact of open wounds with water containing pathogenic agents (12). The United States Centers for Disease Control and Prevention (CDC) estimates that 80,000 cases of vibriosis occur in the United States each year, with approximately 52,000 cases caused by consumption of contaminated seafood (12). Vibriosis can present as a serious infection, with *V. vulnificus* being fatal in *ca.* 20% of reported wound infections and greater than 50% for primary septicemia (12). Given the environmental importance of *Vibrio spp*. in the aquatic ecosystem, namely carbon and nitrogen cycling, eradication of *Vibrio spp*. and the diseases they cause is not possible. Hence, it is essential to understand the environmental parameters associated with their incidence and proliferation to provide early warning for risk of infection, an essential tool for public health and aquaculture.

Altered geographic abundance of *Vibrio spp.* (2, 13) and, notably, the incidence of *V. vulnificus* infections in the eastern United States from 1988 to 2018 (14) show significant northern shifts in the distribution of cases over time. Other investigators have reported similar distributional changes in *V. vulnificus* infections globally that have been linked to climate change (15). Between 1996 and 2010, the majority of cases in the U.S. were caused by *V. parahaemolyticus ca*. 45%, followed by *V. vulnificus ca*. 19%, *V. alginolyticus ca*. 11%, and *V. cholerae* non-O1/non-O139 *ca*. 9% (16). However, infections caused by *V. fluvialis* and *V. mimicus* have increased in recent years (17).

Concerningly, the incidence of cholera and non-cholera *Vibrio spp*. infections has increased in recent decades (18–20), likely linked to changes in the environment, notably temperature and salinity (2, 13), that make regions better suited for the proliferation of *Vibrio spp*. (15). Sea surface temperature (SST) was found to be associated with outbreaks of cholera (21), and warmer water temperatures were correlated with increased numbers of non-cholera *Vibrio spp.* (22). Similarly, sea surface salinity (SSS) was found to be associated with the abundance of *Vibrio spp*., and concerns have been raised with respect to rising sea surface levels and changes in precipitation patterns impacting salinity concentrations, hence the incidence of pathogenic *Vibrio spp.* (19, 22, 23).

Chlorophyll-a (chl-a) has been found to have an association with both cholera (24–26) and non-cholera *Vibrio spp* (27–29). Chl-a concentrations have been used to estimate nutrient loads and phytoplankton blooms, with the latter serving as a proxy for the incidence of zooplankton (21, 30, 31). Since zooplankton, notably copepods, provide a nutrient-rich niche to which *Vibrio spp*. attach (32, 33), chl-a can also serve as a useful indirect indicator of *Vibrio* incidence, when appropriate lag times are considered (34).

In addition, extreme precipitation events have been linked to both cholera (35–37) and non-cholera *Vibrio spp*. infections (38). Anomalous precipitation has been shown to alter the salinity of coastal waters (39), and with extreme precipitation events expected to increase in intensity and frequency with climate change (40), it has been proposed that infections caused by *Vibrio spp*. may further increase in the coming years (18).

Optimal conditions for the growth of *Vibrio spp*. vary depending on genetic composition (9, 41–44). Thus, identifying environmental parameters associated with the incidence and abundance of pathogenic *Vibrio spp*. and identifying those factors associated with the incidence and distribution of *Vibrio* spp. is essential for the development of predictive intelligence and early warning systems. A hypothesis of environmental factors influencing risk of cholera has been proposed (20, 21, 31, 45, 46) and has been employed to mitigate risk of cholera outbreaks in several regions globally (36, 47–50). A few predictive models for non-cholera *Vibrio spp*. have been proposed for regions of the U.S., namely, *V. parahaemolyticus* (51, 52) and *V. vulnificus* (53). Following the proposal of using remote sensing data for investigations of *Vibrio spp.* (31), earth observation satellite products have been used for some predictive models, e.g., *V. parahaemolyticus* in Chesapeake Bay (54), non-cholera *Vibrio spp*. in the Baltic Sea (55), and *V. vulnificus* in some areas of the U.S. (56). Here, the objective was to identify potential environmental parameters associated with incidence and distribution of vibriosis along the eastern seaboard of the U.S. to facilitate development of predictive models for risk assessment.

## MATERIALS AND METHODS

### Vibriosis case data

**Fig 1:**
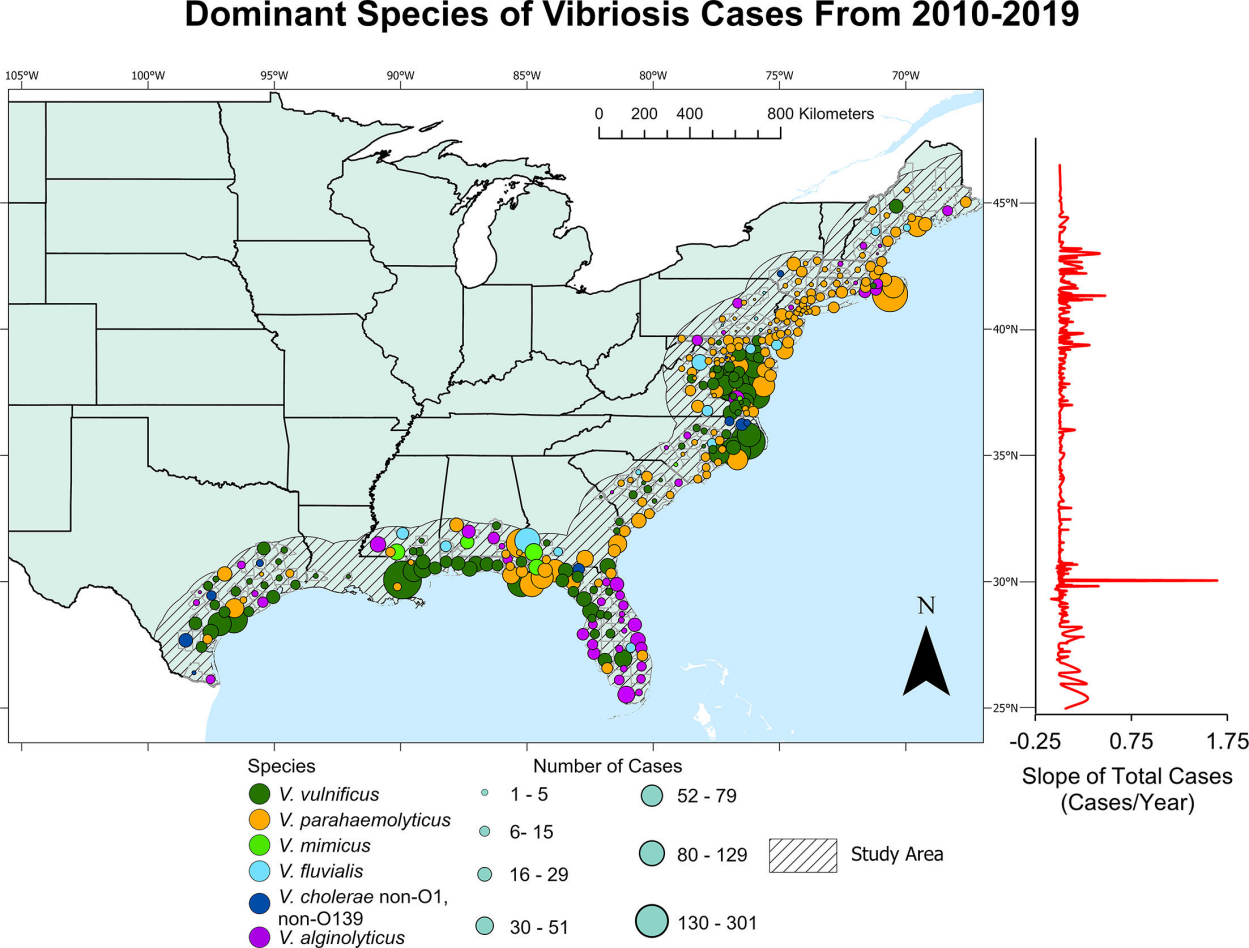
*Vibrio spp*. responsible for most of the vibriosis cases (highest relative abundance) in US counties within 200 km of the US eastern coastline (study area), between 2010 and 2019. The right side of the graph shows changes in the number of cases per year at each latitude point. The base map is from the U.S. Census Bureau and Natural Earth.

Data for reported vibriosis (non-cholera) infections in the U.S. were provided by the CDC Cholera and Other Vibrio Illness Surveillance system (57) and included the number of reported cases between 1990 and 2019, along with the date patients arrived at the hospital, whether the case was confirmed or probable, causative agent, location of the hospital where the case was reported, travel history, and transmission route (foodborne, likely foodborne, nonfoodborne, likely nonfoodborne, or unknown source). For this study, both confirmed and probable cases of vibriosis were considered, but it should be noted that, of the *ca*. 10,000 total cases, only *ca*. 1,000 were categorized as probable cases. Cases in which the patient had traveled recently were omitted from the analysis. Locations of each case were given on varying spatial scales, with some cases reported at the county level, some at the city level, and others at the state level. Hence, state-level cases were not considered because of coarse spatial resolution. City-level cases were converted to the county level, using the county of the identified city for each case. Each case was assigned a latitude and longitude location based on the centroid of each US county where the case was reported. Only cases with assigned latitude and longitude within 200 km of the US east coast (including the Gulf of Mexico) were considered (see Fig. 1 for depiction of the study area).

### Geographical case trend analyses

To observe change in the latitudinal distribution of vibriosis cases over time, an annual trend analysis was conducted as opposed to a smaller time scale because seasonal trends of vibriosis typically show case peaks in the summer months, potentially masking long-term trends. After assigning latitude, the cases were separated by year. The minimum, 25th percentile, median, 75th percentile, and maximum latitude values were computed for the latitudes of all cases in each calendar year. This was done to examine both the general movement of case distributions as well as the extreme extent of case distribution. Once latitude values were computed for each year, Pearson’s correlation coefficient between each percentile and time was computed to determine if there was a significant positive or negative correlation of case latitude over time. The slope of case latitude over time was also computed to determine the magnitude of these changes. These computations were done for all cases of vibriosis, cases of only likely or confirmed foodborne sources, cases of only likely or confirmed nonfoodborne sources, and cases of non-*V*. *cholerae Vibrio spp*., as well as individual *Vibrio spp*., including *V. cholerae* non-O1 and non-O139, *V. alginolyticus*, *V. fluvialis*, *V. mimicus*, *V. parahaemolyticus*, and *V. vulnificus*. For the remaining *Vibrio spp*., there was an insufficient number of reported cases (*ca*. 20) over the 30-year period to perform statistical analyses; hence, they were excluded. Cases for the six *Vibrio spp*. were grouped into 10-year periods (1990–1999, 2000–2009, and 2010–2019), and geographic centroids for each 10-year period were computed for each *Vibrio spp*.

### Environmental parameters

To understand the relationship between long-term geographical trends in vibriosis case data and environmental changes over time, several environmental parameters were selected for analysis. All environmental data were extracted from within 20 km of the US eastern coast (including the Gulf of Mexico) and comprise SST, SSS, chl-a concentration, phytoplankton concentration (separated by size classification), and precipitation. These data were converted to monthly averages prior to subsequent analysis.

SST data were collected from two different sources for 1990–2019. Data for 1990 to July 2002 were collected from the National Oceanic and Atmospheric Administration (NOAA) optimum interpolation sea surface temperature (OISST) data set obtained using the advanced very high resolution radiometer (AVHRR) and visible infrared imaging radiometer suite (VIIRS) (58). Data for August 2002 to 2019 were collected from the Aqua-MODIS daytime 4 km monthly SST data set, using the NASA Ocean Color levels 3 and 4 data browser (59). The Aqua-MODIS data set has a much higher spatial resolution than OISST, but Aqua-MODIS data span only to 2002; hence, two different data sets were used. Data for SSS were from the Ocean Reanalysis System 5 (ORAS5) global ocean reanalysis monthly data set (60). Precipitation data were from ERA5 monthly averaged data sets for total precipitation (61).

Chl-a concentration data were collected from Global Ocean Color (Copernicus-GlobColour), Bio-Geo-Chemical, L4 (monthly and interpolated) from Satellite Observations (1997-ongoing) data set provided by Copernicus Marine Data Store (62). These data were from September 1997 to 2019. Other remote sensing data sources for chl-a concentrations were not readily available prior to this date. This level 4 data set integrates chl-a observations from multiple satellite sensors, including SeaWiFS, MODIS, MERIS, VIIRS (SNPP and JPSS1), and OLCI (Fig. S3A and B), to produce a continuous, blended product improving spatial and temporal accuracy.

Chl-a concentrations can be categorized by phytoplankton size classification. Specifically, microphytoplankton, nanophytoplankton, and picophytoplankton concentrations were calculated from chl-a concentration using several transformation formulas. For this study (63), formulas were used to transform chl-a concentration to the size classification of the phytoplankton. Size classifications of phytoplankton show the highest concentration at different ranges of chl-a, and because chl-a concentration has a non-linear association with *Vibrio spp.* (13), it is important to explore the possibility of relationships within size classification, as they may explain the optimal chl-a concentration range with respect to *Vibrio spp*. abundance. Although other sensors and data sets are available for these environmental parameters, the study reported here prioritized continuity and consistent spatial resolution to enhance accuracy and minimize noise introduced by varying algorithms. The exception was SST, for which the data prior to 2002 were obtained from AVHRR-VIIRS at a coarser 0.25° resolution. Post-2002 data from sensors like MODIS offer finer 4 km resolution. However, previous studies have shown that temperature resolution has little impact on identifying environmental associations and model performance (21, 49). Nonetheless, higher resolution is preferred.

### Environmental parameter trend analysis

To observe variability in selected environmental parameters for the study area throughout the study period, the data were grouped into five multi-year periods: 1990–1996 (except for phytoplankton due to data availability), 1997–2002, 2003–2008, 2009–2014, and 2015–2019. Data were also grouped by season to determine long-term seasonal variability.

Pearson’s correlation coefficients were calculated between minimum, 25th percentile, median, 75th percentile, and maximum values for each parameter to assess the direction and significance of temporal trends. Slopes were also computed to quantify the rate and magnitude of change over time. In summary, this approach provides a standardized method to evaluate both annual and seasonal shifts in environmental conditions across the eastern and southern U.S. coastal study area.

### Extreme gradient boosting modeling

To determine the relative influence and complex relationships of each environmental parameter on reported vibriosis cases, eXtreme Gradient Boosting (XGBoost) Models were used. XGBoost is a machine learning method that uses gradient-boosted decision trees (64). Environmental data were associated with each data point by using the average value from the five data points closest to the location. Environmental data were lagged by 1 month, as there is likely to be a lagged impact of environmental variables on reported cases. Location data points were selected using all counties within 200 km of the U.S. eastern coastline that had reported a case of vibriosis for causative species, including *V. cholerae* non-O1 and non-O139, *V. alginolyticus*, *V. fluvialis*, *V. mimicus*, *V. parahaemolyticus*, and *V. vulnificus*. Each data point was assigned a categorical variable indicating vibriosis case presence or absence. Therefore, each data point included a specific location, environmental data (1-month lag) associated with that location, and an indicator of case presence or absence for that month and location. Data from October 1997 (the earliest available data for chl-a/phytoplankton) to December 2019 were used.

The data had an imbalanced class distribution, with the presence class being a minority and highly imbalanced for all *Vibrio spp*. (~99 absence data points to every 1 presence data point). This bias in data sets can affect the XGBoost model, potentially leading to ignoring the minority class entirely. To address this problem, two approaches used were to randomly resample the imbalanced data set: undersampling, which involves deleting some data from the majority class (absence), and oversampling, which involves duplicating data from the minority class (presence). Given that our minority class was significantly smaller than the majority class, we opted for random undersampling, ensuring equal representation of each class. This is preferable to random oversampling of the minority class because random oversampling can lead to model overfitting and a misrepresentation of model performance under real predictive scenarios. This preprocessing step was crucial for unbiased model training. The *imbalanced-learn* () library was used for RandomUnderSampler. As random undersampling can have large differences in the majority class data points selected, impacting model fitting and results, random undersampling was performed 50 times for each *Vibrio sp*. data set, thus providing data for 50 models for each *Vibrio sp*. The use of multiple iterations assists in providing clarity on model performance across most of the data set and reduces the majority class selection bias.

In establishing the framework for the XGBoost model, six environmental parameters were selected as input features, with chl-a excluded from the input features. This was to reduce the impact of collinearity and overfitting since phytoplankton data were also included. The binary classification of vibriosis presence served as the response variable. Each data set was partitioned into two subsets using the train_test_split function from the sklearn.model_selection module: 70% allocated for training and 30% for testing. The classification model was constructed using the XGBClassifier function from the XGBoost library. Initial settings for the model parameters included the number of estimators, learning rate, maximum depth of trees, subsample ratio, and column sample by tree fraction. To refine the parameters, GridSearchCV imported from the sklearn.model_selection was employed to conduct hyperparameter tuning, aiming to optimize model settings. The grid search emphasized maximizing accuracy and incorporated fivefold cross-validation to validate model performance across different subsets of data. The best-performing model was determined based on the results of this hyperparameter tuning, ensuring the selected model offered the most effective combination of parameters for generalizability and predictive accuracy.

In the model evaluation phase, the performance of the best model obtained from the GridSearchCV was assessed using several key metrics to determine effectiveness on the testing data set. These metrics included accuracy, precision, recall, F1 score, and area under the curve (AUC) score. SHapley Additive exPlanations (SHAP) values proposed by Lundberg and Lee (65) were used to explain the physical interpretation of model outputs. Utilizing the game-theoretic approach of Shapley values, SHAP provides a detailed decomposition of the predictive contribution of each environmental parameter. This approach allowed both global and local interpretability. Globally, SHAP reveals the overall importance and effect of each feature across all predictions, helping to identify key environmental drivers of vibriosis presence. Locally, it explains the contribution of each feature to individual predictions, offering insight into specific cases that may be critical for targeted environmental or public health intervention. Fifty XGBoost models were created for each species (*V. cholerae*, non-O1 and non-O139, *V. alginolyticus*, *V. fluvialis*, *V. mimicus*, *V. parahaemolyticus*, and *V. vulnificus*), in addition to total *Vibrio* spp. infections, using three case groupings: foodborne only, nonfoodborne only, and all cases combined.

### K-means clustering analysis

As a complementary analysis to better understand the environmental conditions under which vibriosis cases were reported, k-means clustering analysis was conducted. For this analysis, vibriosis cases were divided according to individual *Vibrio spp*. (including *V. cholerae*, non-O1 and non-O139, *V. alginolyticus*, *V. fluvialis*, *V. mimicus*, *V. parahaemolyticus*, and *V. vulnificus*) and further divided into foodborne and nonfoodborne cases. K-means clustering was performed in Minitab for three centroids in each analysis. Results for different numbers of centroids did not provide greater physical clarity with respect to environmental conditions associated with cases and using the same number of centroids for each group made direct comparison easier, particularly for foodborne and nonfoodborne cases caused by the same *Vibrio spp*. Environmental data were associated with each case just as they were in the XGBoost models. Monthly cases and environmental data were used, beginning with October 1997 to December 2019. Only data points with vibriosis case presence were used in the analyses.

## RESULTS

### Geographical case trends

**Fig 2:**
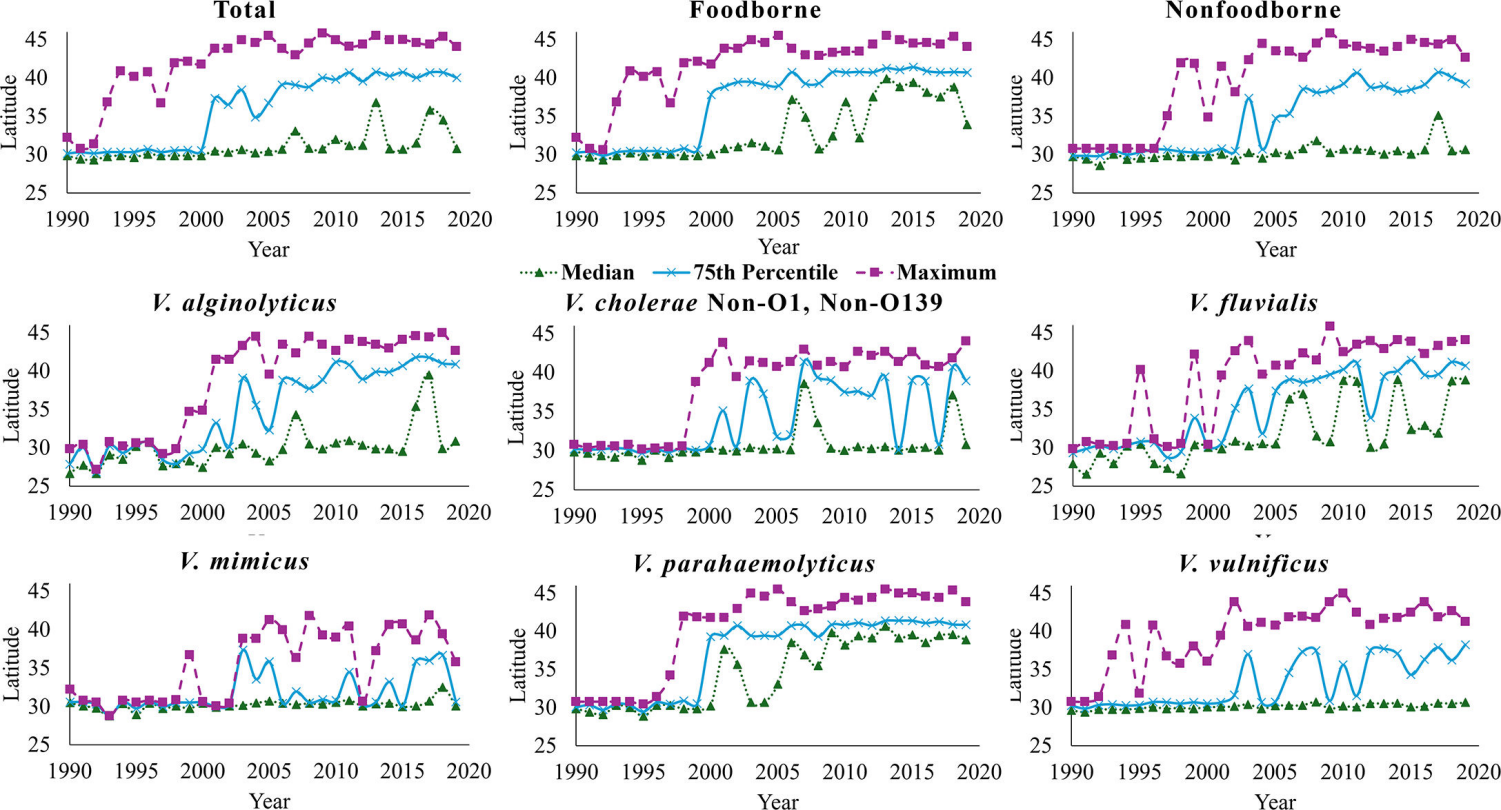
Median, 75th percentile, and maximum latitude of vibriosis cases within 200 km of the eastern US coastline and the Gulf of Mexico, 1990–2019.

Latitudinal shifts in vibriosis cases over time, including median, 75th percentile, and maximum latitude, are shown in Fig. 2. Correlation coefficient and slope with time for minimum, 25th percentile, median, 75th percentile, and maximum latitude are provided in Table 1. Interestingly, the minimum latitude for the reported causative agent of vibriosis, except *V. vulnificus*, decreased over time, suggesting a southernmost latitude shift southward. All case types (foodborne, nonfoodborne, cholera, non-cholera, and causative agent) showed positive correlation and slope for the remaining values. All values were statistically significant at 99% confidence level, except the 25th percentile for nonfoodborne, *V. alginolyticus*, and *V. mimicus* cases and median for *V. cholerae* non-O1, non-O139 cases.

**TABLE 1:** Correlation coefficients and slopes of minimum, 25th percentile, median, 75th percentile, and maximum latitude of vibriosis cases within 200 km of the eastern US coastline and the Gulf of Mexico, 1990–2019^*a*^

Species/infection type	Total					Foodborne				
Percentile	Minimum	25th Percentile	Median	75th Percentile	Maximum	Minimum	25th Percentile	Median	75th Percentile	Maximum
Correlation	−0.373	**0.822**	**0.658**	**0.920**	**0.782**	−0.153	**0.786**	**0.831**	**0.885**	**0.769**
Slope (km/year)	−1.73	8.86	14.98	52.28	42.19	−0.89	10.61	38.69	52.93	40.95
Species/infection type	Non-*V*. *cholerae*					Nonfoodborne				
Percentile	Minimum	25th Percentile	Median	75th Percentile	Maximum	Minimum	25th Percentile	Median	75th Percentile	Maximum
Correlation	−0.373	**0.802**	**0.784**	**0.925**	**0.790**	**−0.440**	0.408	**0.585**	**0.910**	**0.852**
Slope (km/year)	−1.73	8.79	22.27	53.25	42.51	−2.40	3.42	8.11	49.55	61.38
Species/infection type	*V. cholerae* Non-O1, Non-O139					*V. alginolyticus*				
Percentile	Minimum	25th Percentile	Median	75th Percentile	Maximum	Minimum	25th Percentile	Median	75th Percentile	Maximum
Correlation	−0.062	**0.686**	0.380	**0.693**	**0.822**	−0.403	0.243	**0.605**	**0.918**	**0.878**
Slope (km/year)	−1.03	8.41	10.16	37.59	54.61	−6.35	3.34	19.76	61.55	70.30
Species/infection type	*V. fluvialis*					*V. mimicus*				
Percentile	Minimum	25th Percentile	Median	75th Percentile	Maximum	Minimum	25th Percentile	Median	75th Percentile	Maximum
Correlation	−0.074	**0.659**	**0.716**	**0.891**	**0.823**	−0.196	0.145	**0.478**	**0.477**	**0.727**
Slope (km/year)	−1.01	14.12	35.31	52.07	60.22	−3.56	1.61	3.72	14.40	42.11
Species/infection type	*V. parahaemolyticus*					*V. vulnificus*				
Percentile	Minimum	25th Percentile	Median	75th Percentile	Maximum	Minimum	25th Percentile	Median	75th Percentile	Maximum
Correlation	−0.305	**0.719**	**0.890**	**0.867**	**0.835**	0.197	**0.581**	**0.798**	**0.801**	**0.767**
Slope (km/year)	−2.12	14.73	49.65	54.13	61.01	1.45	5.07	3.40	32.14	39.29

^*a*^ Bolded correlations are statistically significant (*P* < 0.01).

For the reported total, foodborne, and non-*V*. *cholerae*, the 75th percentile latitude shifted most, with reported foodborne cases greatest, 0.477° (~52.9 km) per year. For others, the northernmost latitude shifted most over time, the largest being 0.633° (~70.3 km) per year for *V. alginolyticus* and the least being 0.354° (~39.3 km) for *V. vulnificus*.

**Fig 3:**
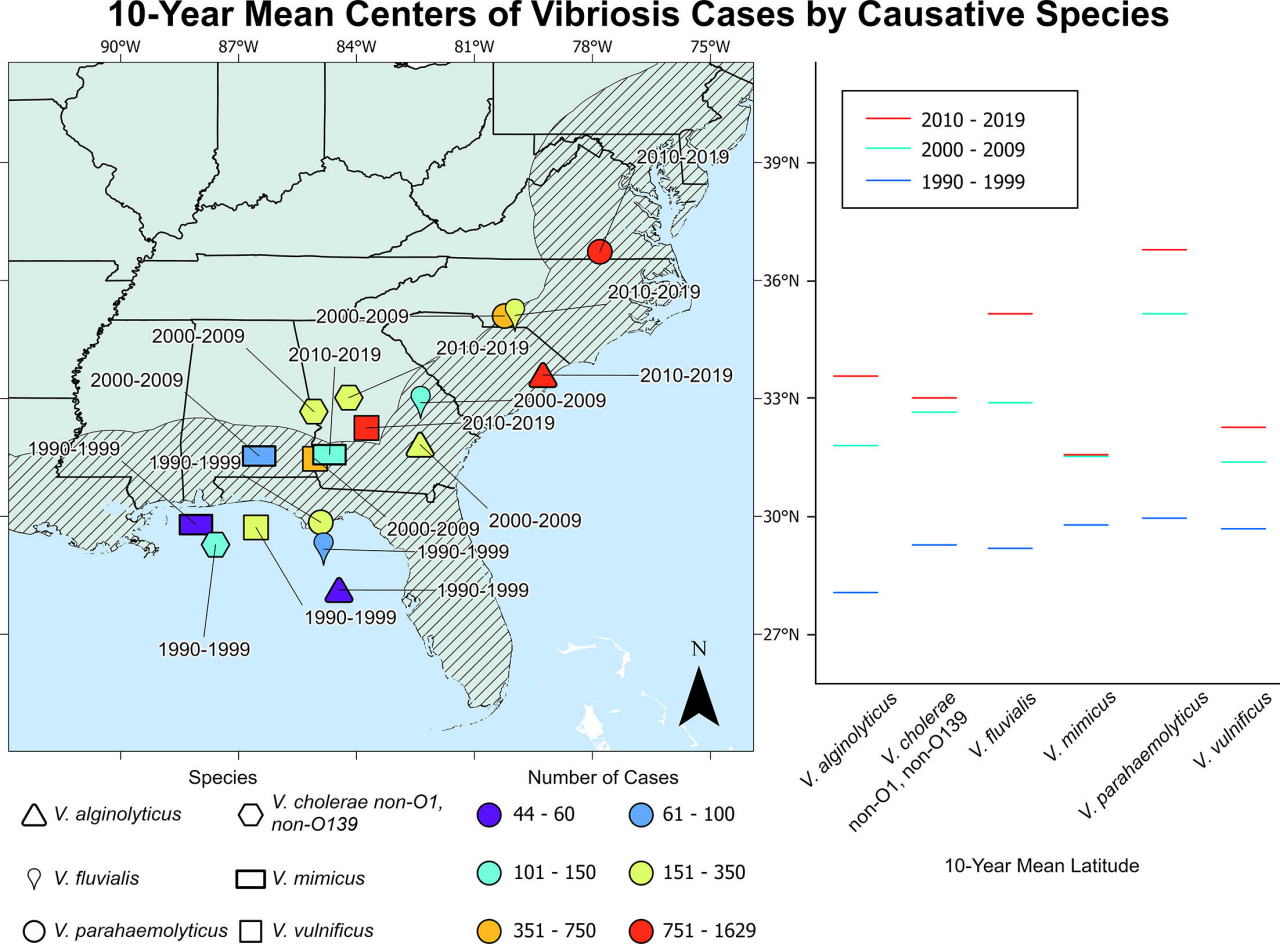
Ten-year mean geographic centroids of vibriosis cases by *Vibrio spp*. identified as the causative agent, 1990–2019. The number of cases represents the total number of cases for each 10-year period. Bars on the right are latitude markers for each 10-year period. The base map is from the U.S. Census Bureau and Natural Earth.

**Fig 4:**
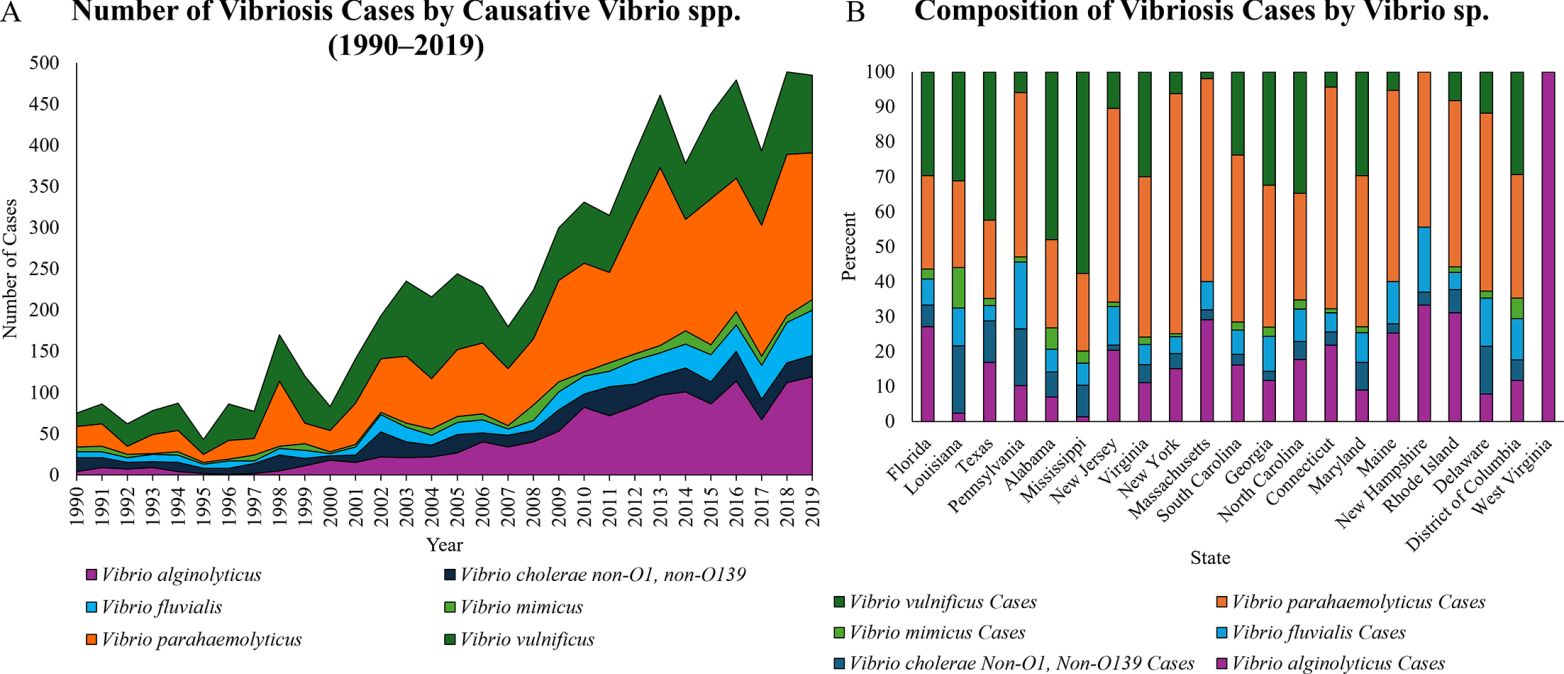
(**A**) Vibriosis cases over time separated by causative agent (*Vibrio sp*.). Data obtained from records of counties located within 200 km of the eastern coastline and the Gulf of Mexico, 1990–2019. (**B**) Composition of vibriosis cases by *Vibrio sp*. responsible for infection, presented by state using data from counties within 200 km of the eastern coastline and the Gulf of Mexico, 1990–2019. Vibriosis cases caused by other *Vibrio spp*. are not included.

Figure 3 presents results for 10-year geographic centroids, with the least total geographic shift over the last three decades for *V. mimicus* and the most for *V. alginolyticus*, *V. fluvialis*, and *V. parahaemolyticus*. Results show case volume, by color scale, with increases noted for all species, but especially *V. parahaemolyticus*, *V. vulnificus*, and *V. alginolyticus*. For clarity on case distribution, Fig. 4 presents the relative abundance of vibriosis cases for each state along the U.S. eastern seaboard and number of vibriosis cases between 1990 and 2019.

### Environmental parameter trends

Box and whisker plots of environmental parameters for multi-year periods between 1990 and 2019 are provided in the supplemental material. In general, SST and SSS have significantly increased along the U.S. eastern and southern seaboard between 1990 and 2019. Other parameters did not show consistent or statistically significant trends across all seasons. However, some exhibited notable seasonal variability over the past few decades. Notably, picophytoplankton median and 75th percentile values significantly (*P* < 0.05) decreased during summer and fall (i.e., seasons associated with high vibriosis case reports) from 1997 to 2019. Also, SST slopes for the 25th, median, and 75th percentile values were greatest in the summer. Annual correlation coefficient and slope with time for minimum, 25th percentile, median, 75th percentile, and maximum latitude are provided in Table 2.

**TABLE 2:** K-means clustering analysis results^*a*^^,^^*b*^

Foodborne									Nonfoodborne							
*V. alginolyticus*																
Variable	SSS (ppt)	SST (°C)	Chl-a (mg/L)	Micro (mg/L)	Pico (mg/L)	Nano (mg/L)	Precip (mm)	Observations (*N*)	SSS (ppt)	SST (°C)	Chl-a (mg/L)	Micro (mg/L)	Pico (mg/L)	Nano (mg/L)	Precip (mm)	Observations (*N*)
Cluster 1	25.4	23.5	16.52	16.51	0	0.02	3	39	28.6	26.7	16.58	16.56	0	0.02	3.3	192
Cluster 2	31.3	18.6	9.58	9.36	0.01	0.21	3.2	59	31.2	16.5	8.91	8.64	0.02	0.25	3.3	207
Cluster 3	35.6	26.3	3.79	3.28	0.14	0.36	2.6	58	35.5	27	3.83	3.33	0.15	0.35	3	433
Grand centroid	31.4	22.7	9.16	8.89	0.06	0.22	2.9	156	32.9	24.3	8.04	7.7	0.08	0.25	3.1	832
Q1–Q3	30.4–35.6	19.1–27.8	4.13–13.13	3.54–12.99	0.00–0.09	0.03–0.40	1.8–4.0	N/A	31.4–36.0	21.4–28.9	3.01–11.69	2.36–11.62	0.00–0.15	0.05–0.41	1.5–4.1	N/A
V. cholerae non-O1, non-O139																
Variable	SSS (ppt)	SST (°C)	Chl-a (mg/L)	Micro (mg/L)	Pico (mg/L)	Nano (mg/L)	Precip (mm)	Observations (*N*)	SSS (ppt)	SST (°C)	Chl-a (mg/L)	Micro (mg/L)	Pico (mg/L)	Nano (mg/L)	Precip (mm)	Observations (*N*)
Cluster 1	28.3	19	10.57	10.4	0	0.18	3.3	130	32	26.7	14.93	14.9	0	0.02	3.4	40
Cluster 2	34.6	26.8	4.78	4.28	0.12	0.38	2.9	81	33.9	26.3	5.38	4.97	0.07	0.33	3	31
Cluster 3	20	26.6	16.34	16.32	0	0.02	4.2	68	18.9	27.2	14.66	14.6	0	0.06	5.1	26
Grand centroid	28.1	23.1	10.3	10.06	0.04	0.2	3.4	279	29.1	26.7	11.81	11.65	0.03	0.13	3.7	97
Q1–Q3	22.6–34.3	18.4–28.1	6.17–13.84	5.69–13.84	0.00–0.00	0.00–0.37	1.5–4.4	N/A	23.3–34.6	23.9–30.3	8.15–15.39	7.87–15.39	0.00–0.00	0.00–0.25	1.6–4.9	N/A
*V. fluvialis*																
Variable	SSS (ppt)	SST (°C)	Chl-a (mg/L)	Micro (mg/L)	Pico (mg/L)	Nano (mg/L)	Precip (mm)	Observations (*N*)	SSS (ppt)	SST (°C)	Chl-a (mg/L)	Micro (mg/L)	Pico (mg/L)	Nano (mg/L)	Precip (mm)	Observations (*N*)
Cluster 1	24.1	26.1	17.79	17.78	0	0.01	3.6	81	19.9	25.8	13.09	13.01	0	0.08	3.7	28
Cluster 2	28.6	17.3	10.01	9.82	0.01	0.18	3.1	124	32.7	26.2	14.15	14.11	0	0.04	3.7	31
Cluster 3	34.3	26.2	5.86	5.47	0.08	0.32	3.2	125	33.8	21.4	5.3	4.85	0.1	0.35	3.1	47
Grand centroid	29.7	22.8	10.35	10.13	0.03	0.19	3.2	330	29.8	24	9.95	9.71	0.05	0.19	3.4	106
Q1–Q3	25.7–35.0	18.7–27.8	6.51–13.68	6.06–13.65	0.00–0.00	0.00–0.35	1.9–4.4	N/A	23.8–35.4	21.4–28.8	5.90–13.08	5.41–13.06	0.00–0.01	0.00–0.34	2.0–4.0	N/A
Foodborne									Nonfoodborne							
*V. mimicus*																
Variable	SSS (ppt)	SST (°C)	Chl-a (mg/L)	Micro (mg/L)	Pico (mg/L)	Nano (mg/L)	Precip (mm)	Observations (*N*)	SSS (ppt)	SST (°C)	Chl-a (mg/L)	Micro (mg/L)	Pico (mg/L)	Nano (mg/L)	Precip (mm)	Observations (*N*)
Cluster 1	32.8	25.5	15.59	15.57	0	0.028	3.8	20	32.1	28.5	15.18	15.17	0	0.015	3.6	13
Cluster 2	21.1	24.9	12.82	12.74	0	0.088	4.1	51	20.7	24.8	11.27	11.17	0	0.101	4.7	15
Cluster 3	33.5	21.2	5.27	4.79	0.073	0.408	3.2	47	34	23.3	6.77	6.44	0.048	0.284	1.9	16
Grand centroid	28	23.5	10.29	10.05	0.029	0.205	3.7	118	28.9	25.3	10.79	10.63	0.018	0.142	3.4	44
Q1–Q3	22.0–34.5	20.2–28.6	6.01–13.04	5.52–12.95	0.00–0.00	0.00–0.40	2.3–4.5	N/A	22.7–35.0	22.4–29.4	8.77–14.23	8.54–14.23	0.00–0.00	0.00–0.21	1.1–3.8	N/A
*V. parahaemolyticus*																
Variable	SSS (ppt)	SST (°C)	Chl-a (mg/L)	Micro (mg/L)	Pico (mg/L)	Nano (mg/L)	Precip (mm)	Observations (*N*)	SSS (ppt)	SST (°C)	Chl-a (mg/L)	Micro (mg/L)	Pico (mg/L)	Nano (mg/L)	Precip (mm)	Observat-ions (*N*)
Cluster 1	14.9	22	18.55	18.55	0	0.01	3.7	192	15.2	25.5	17.84	17.83	0	0.02	3.3	122
Cluster 2	31.6	20.9	6.93	6.56	0.05	0.31	3.3	656	34.1	25	4.81	4.31	0.1	0.4	2.9	312
Cluster 3	31.1	22.2	15.98	15.97	0	0.02	3.4	396	31.2	24.1	13.22	13.15	0	0.07	3.4	312
Grand centroid	28.9	21.5	11.6	11.4	0.03	0.17	3.4	1244	29.8	24.7	10.46	10.22	0.05	0.2	3.2	746
Q1–Q3	27.9–33.5	18.0–25.5	7.12–15.45	6.74–15.45	0.00–0.00	0.00–0.35	2.0–4.3	N/A	28.4–35.2	21.2–29.1	5.56–14.38	5.05–14.37	0.00–0.04	0.00–0.39	1.7–4.2	N/A
*V. vulnificus*																
Variable	SSS (ppt)	SST (°C)	Chl-a (mg/L)	Micro (mg/L)	Pico (mg/L)	Nano (mg/L)	Precip (mm)	Observations (*N*)	SSS (ppt)	SST (°C)	Chl-a (mg/L)	Micro (mg/L)	Pico (mg/L)	Nano (mg/L)	Precip (mm)	Observations (*N*)
Cluster 1	31.9	24.8	12.85	12.78	0	0.065	3.5	119	28.7	28.1	12.85	12.79	0	0.06	3.9	381
Cluster 2	34.9	26	4.2	3.68	0.13	0.392	3.1	135	34.6	26.9	5.04	4.56	0.11	0.37	3.8	323
Cluster 3	18.2	25	14.95	14.89	0	0.056	4	81	14.1	25.7	22.27	22.27	0	0	3.2	137
Grand centroid	29.8	25.3	9.87	9.63	0.05	0.2	3.5	335	28.6	27.3	11.38	11.17	0.04	0.17	3.7	841
Q1–Q3	25.0–35.3	22.3–29.6	5.08–13.54	4.52–13.54	0.00–0.06	0.00–0.38	1.8–4.6	N/A	22.3–35.3	25.0–30.5	6.61–14.79	6.19–14.79	0.00–0.01	0.00–0.35	2.1–4.9	N/A

^*a*^ The left side of the table includes clusters for foodborne cases and the right side for nonfoodborne cases. The grand centroid is the overall center for each dataset and Q1–Q3 the range of values between the 25th percentile to the 75th percentile.

^*b*^ “N/A” indicates the number of observations that are not provided for the interquartile range (Q1-Q3) because the values are a range, not a single value.

### Extreme gradient boosting model results

**Fig 5:**
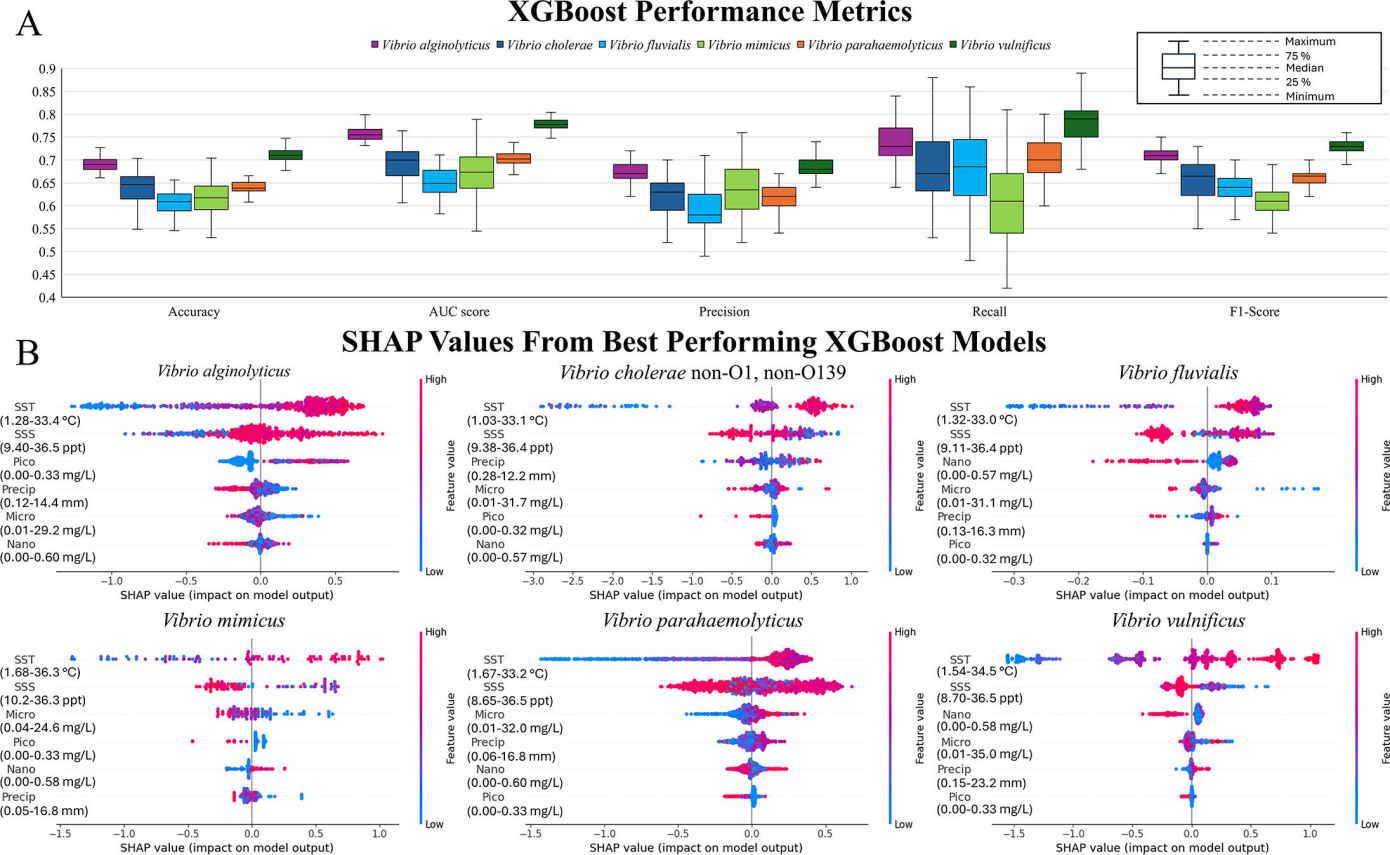
(**A**) Box and whisker plots of performance metrics for XGBoost models. Medians are inclusive. Outliers are not excluded. (**B**) SHAP values for XGBoost models with the highest accuracy for each *Vibrio sp*. Environmental variables are listed in order of importance for model results, with the top variable being the highest relative importance and the bottom variable the least relative importance. Ranges of environmental values for the testing data sets are provided below each variable.

Model performance results for the test samples are shown in Fig. 5A. For some *Vibrio spp*., models using only nonfoodborne cases showed modest improvements compared to models using all cases. However, these improvements were inconsistent across species, and performance metrics became more variable, likely due to the reduced number of data points in the subset models. As a result, only models trained using total case data are evaluated in the main text. A detailed comparison of model performance for foodborne, nonfoodborne, and total cases is provided in the supplemental material.

For models exploring the total incidence of reported vibriosis, the average and median accuracy for all 50 iterations of the models were between 60.9% and 71.0%. The range of accuracies was largest for *V. mimicus* (0.53–0.72), likely because *V. mimicus* had fewest reported cases, therefore least data points for random undersampling, leading to greater variety in majority class data selection. *V. mimicus* models showed the largest range of results for other performance metrics, as did the *V. cholerae* non-O1, non-O139 and *V. fluvialis* models, the two other species showing relatively lower reported cases. In contrast, models for *V. alginolyticus*, *V. parahaemolyticus*, and *V. vulnificus* had smaller ranges of performance statistics, likely due to larger minority classes. The best-performing model was *V. vulnificus* by all performance metrics, followed by *V. alginolyticus*. The other models yielded mixed rankings among the performance metrics, but *V. mimicus* models had relatively low recall and F1 score, while *V. fluvialis* models showed both low AUC scores and low precision. Most models had fewer false negatives than false positives (recall was greater than precision) apart from *V. mimicus*. This is beneficial, as cases are very likely underreported, and the presence of *Vibrio spp*. in the aquatic environment does not necessarily lead to infection. Thus, slightly overpredicting vibriosis case presence and having more false positives should be expected and is preferable to false negatives, which could be more dangerous to public health if the model is used for future prediction and mitigation.

Figure 5B depicts SHAP values for the models with the highest accuracy for each *Vibrio sp*., along with environmental parameters listed in order of relative importance to the model output. Variation was noted among SHAP values, relationships between environmental variables, and ranking of variable importance for each iteration of the models, but the best performing were selected as they were best able to model results between environmental parameters and vibriosis, warranting the closest examination compared to other model results. Unfortunately, it was not possible to compile the model iterations into one result with SHAP values, as they are based on the testing data sets used, which is dependent on random undersampling. However, trends among model iterations are captured in Fig. 5B and explored further.

A positive SHAP value indicated a feature had a positive impact on case presence in the model results, while a negative SHAP value indicates a feature had a negative impact on case presence. Higher absolute SHAP values indicate more influence on model outputs. A high feature value indicated a high value for that variable within the context of the data set, while a low feature value indicated a low value for that variable. For example, an SSS point with a positive SHAP value and low feature value would indicate that low SSS contributed to the presence of a case in the model. The range of environmental values in the testing results is provided in Fig. 5B for clarity on high and low feature values.

SST and SSS were considered the most important variables in the model results, with very few model iterations deviating from this trend. Ranking of relative importance for the remaining variables varied from model to model. SST had a general positive linear relationship with SHAP values, with increasing SST leading to increased prediction of vibriosis. All other variables experienced a nonlinear relationship with SHAP values for all models, with only a few exceptions.

For *V. alginolyticus*, the highest contribution to vibriosis presence was consistent with high SST (~33°C), high SSS (~36.5 ppt), moderate-to-high picophytoplankton (~0.16–0.33 mg/L), low precipitation (~0.12 mm), low microphytoplankton (~0.01 mg/L), and a large range of nanophytoplankton. The highest contribution to vibriosis absence was consistent, low SST (~1.3°C), low SSS (~9.4 ppt), low picophytoplankton (~0 mg/L), high precipitation (~14.4 mm), a large range of microphytoplankton, and high nanophytoplankton (~0.60 mg/L). Highest SSS values provided the most contribution to presence, while the lowest SST values had the highest contribution to absence.

For *V. cholerae* non-O1, non-O139, highest contributions to vibriosis occurrence were consistent, with high SST (~33°C), low SSS (~9.4 ppt), and a wide range of precipitation values. Highest contributions to vibriosis absence were low SST values (slightly above ~1.3°C), high SSS (~36.4 ppt), a large range of precipitation values, high microphytoplankton (~31.7 mg/L), and high picophytoplankton (~0.32 mg/L). Other contributions to presence and absence by other variables were either small or for very few data points. High and low SST values had the highest contribution to presence and absence.

For *V. fluvialis*, the highest contribution to vibriosis occurrence was consistent with high SST (slightly below ~33°C), moderate SSS (~23 ppt), low-to-moderate nanophytoplankton (~0–0.28 mg/L), and low microphytoplankton (~0.01 mg/L). Highest contributions to vibriosis absence were low SST values (~1.3°C), high SSS (~36.4 ppt), high nanophytoplankton (~0.57 mg/L), high microphytoplankton (~31.1 mg/L), and a large range of precipitation values. Other contributions to presence and absence by other variables were either small or for very few data points. Low microphytoplankton values had the highest contribution to occurrence, though for only a few data points; otherwise, high SST values and moderate SSS values and low SST values had the highest contribution to absence.

For *V. mimicus*, the highest contributions to vibriosis occurrence were consistent with high SST (~36°C), moderate SSS (~23 ppt), low microphytoplankton (~0.04 mg/L), low picophytoplankton (~0 mg/L), and high nanophytoplankton (~0.58 mg/L). Highest contributions to vibriosis absence were low SST values (~1.7°C), high SSS (~36.4 ppt), high microphytoplankton (~24.6 mg/L), high picophytoplankton (~0.33 mg/L), and low nanophytoplankton (~0.00 mg/L). Other contributions to the presence and absence by other variables were either small or had very few data points. High SST values had the highest contribution to occurrence, and low SST values had the highest contribution to absence.

For *V. parahaemolyticus*, the highest contributions to vibriosis occurrence were consistent with high SST (slightly below ~33°C), moderate SSS (slightly above ~23 ppt and slightly below ~36 ppt), high microphytoplankton (~32.0 mg/L), high precipitation (slightly below ~16.8 mm), and high nanophytoplankton (~0.60 mg/L). Highest contributions to vibriosis absence were low SST values (~1.7°C), high SSS (~36.5 ppt), low microphytoplankton (~0.01 mg/L), low precipitation (~0.06 mm), high nanophytoplankton (slightly below ~0.60 mg/L), and high picophytoplankton (~0.33 mg/L). Other contributions to occurrence and absence by other variables were either small or had very few data points. Moderate SSS values had the highest contribution to presence, and low SST values had the highest contribution to absence.

For *V. vulnificus*, the highest contributions to vibriosis occurrence were consistent with high SST (~34°C), low SSS (~8.7 ppt), large range of microphytoplankton, and high precipitation (~23.2 mm). Highest contributions to vibriosis absence were low SST values (~1.5°C), high SSS (slightly below ~36.5 ppt), high nanophytoplankton (~0.58 mg/L), large range of microphytoplankton, low precipitation (~0.15 mm), and high picophytoplankton (~0.33 mg/L). Other contributions to presence and absence by other variables were either small or had very few data points. High SST values had the highest contribution to presence, and low SST values had the highest contribution to absence.

### K-means clustering results

**Fig 6:**
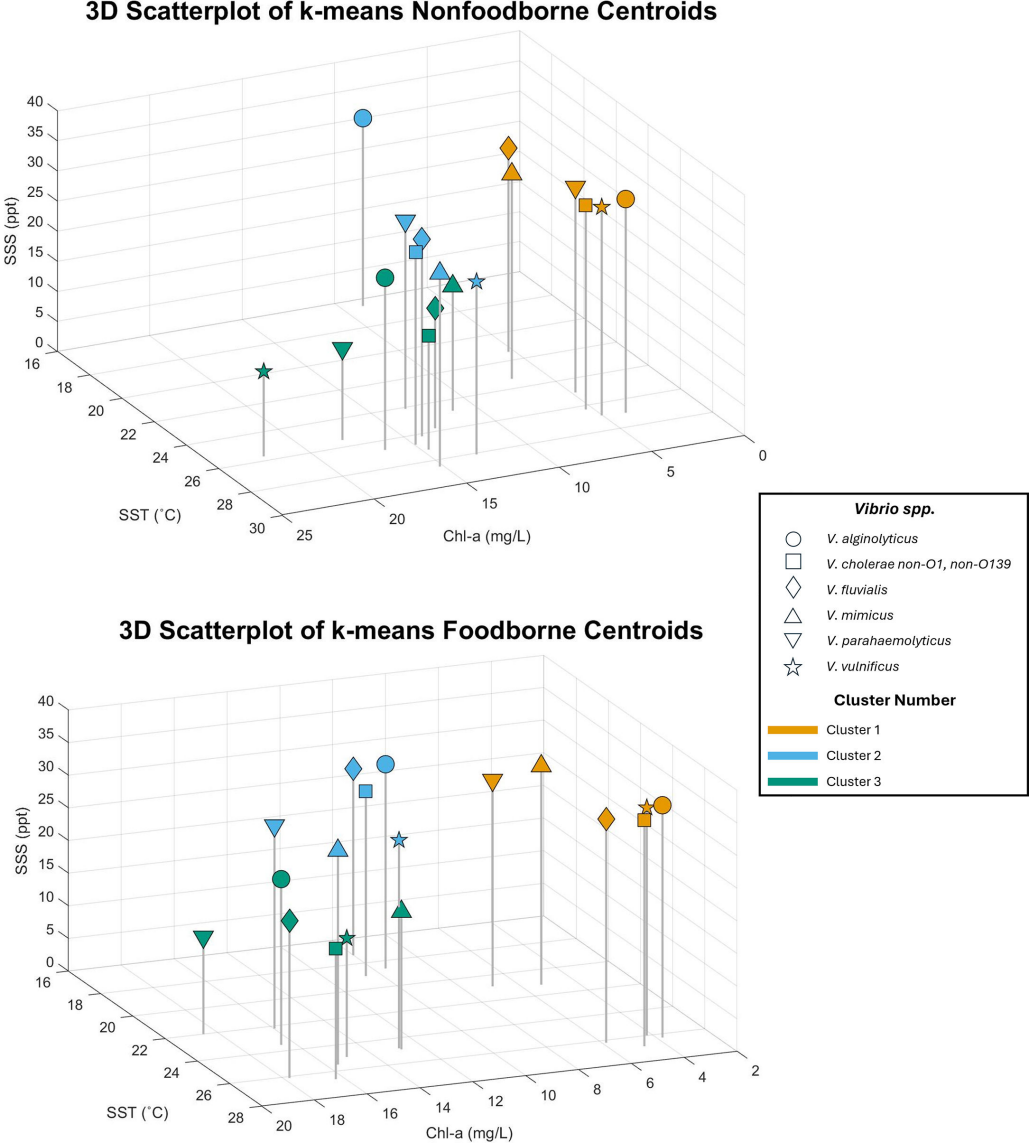
3D scatterplots of SST, SSS, and chl-a from k-means centroids.

Table 3 displays results of k-means clustering analysis for all *Vibrio spp*., separated by foodborne and nonfoodborne. Most had three distinct clusters, as illustrated by Fig. 6. Generally, one with SSS ~30–35 ppt, SST ~24°C–27°C, and chl-a concentration ~4–7 mg/L, and a second cluster with slightly lower SSS, slightly different SST (some higher, some lower), a higher chl-a concentration of ~10–15 mg/L, and higher precipitation. The third generally had SSS of ~15–20 ppt, SST of ~22°C–27 °C, and chl-a concentrations of ~12–22 mg/L. The three clusters were consistent for all *Vibrio spp*. and both foodborne and nonfoodborne cases. However, the distribution of cases within the clusters (i.e., the number of cases assigned to each cluster) was inconsistent between *Vibrio spp*. and foodborne and nonfoodborne cases, indicating that, while these are patterns for environmental conditions, cases are reported at different frequencies within these conditions, depending on species and infection mechanism. Additionally, differences in environmental values between foodborne and nonfoodborne clusters, while noted, were small and inconsistent across *Vibrio spp*., with the only consistency being nonfoodborne infections having generally higher SST (~1°C–3°C for all species) and SSS (~1 ppt in four out of six species) compared to foodborne infections. Therefore, both foodborne and nonfoodborne cases appear to be associated with similar environmental conditions.

**TABLE 3:** Correlation coefficients and slopes of minimum, 25th percentile, median, 75th percentile, and maximum values of environmental variables for multi-year periods (1990–1996, 1997–2002, 2003–2008, 2009–2014, and 2015–2019) within 20 km of the eastern and southern US coastline^*a*^^,^^*b*^

Parameter	SST				
Percentile	Minimum	25th Percentile	Median	75th Percentile	Maximum
Correlation	−0.547	0.010	**0.919**	**0.947**	**0.864**
Slope (°C/period)	−0.272	0.002	0.183	0.471	2.638
Parameter	SSS				
Percentile	Minimum	25th Percentile	Median	75th Percentile	Maximum
Correlation	**0.891**	**0.985**	**0.951**	**0.965**	**0.929**
Slope (ppt/period)	0.521	0.184	0.254	0.110	0.051
Parameter	Precipitation				
Percentile	Minimum	25th Percentile	Median	75th Percentile	Maximum
Correlation	−0.757	0.371	0.577	0.771	0.547
Slope (dm^3^/period)	−0.02	0.04	0.07	0.11	1.45
Parameter	Microphytoplankton				
Percentile	Minimum	25th Percentile	Median	75th Percentile	Maximum
Correlation	−0.007	**0.906**	0.834	0.751	0.855
Slope (mg/L/period)	0.000	0.138	0.285	0.633	1.773
Parameter	Picophytoplankton				
Percentile	Minimum	25th Percentile	Median	75th Percentile	Maximum
Correlation	N/A	N/A	−0.831	−0.884	**−0.908**
Slope (mg/L/period)	0.000	0.000	−0.033	−0.006	−1.24E-12
Parameter	Nanophytoplankton				
Percentile	Minimum	25th Percentile	Median	75th Percentile	Maximum
Correlation	N/A	−0.702	−0.470	−0.332	0.528
Slope (mg/L/period)	0.000	−0.030	−0.002	−0.002	5.03E-07

^*a*^ Bolded correlations are statistically significant (*P* < 0.05).

^*b*^ “N/A” indicates the number of observations that are not provided for the interquartile range (Q1-Q3) because the values are a range, not a single value.

## DISCUSSION

Observed environmental changes, including increased coastal water temperatures in the Gulf of Maine and Middle Atlantic Bight (66), frequency and severity of marine heatwaves along the northeastern United States (67), salinity variability in the upper ocean (68), precipitation extremes (69), and chlorophyll concentrations in coastal regions (70), coincide with results obtained in the study, namely significant increases to coastal SST and SSS and northern shift of the incidence of *Vibrio spp*. infections occurring in the population living along the eastern U.S. coastline as reported for 1990–2019. Determination of the latitudinal shift of *V. vulnificus* supports the findings reported by Archer et al. (14). Interestingly, latitudinal increase in the northern case limit for *V. vulnificus* over time, while significant, was lowest of all *Vibrio spp*., at 0.354 degrees (~39.3 km) per year for northernmost reported case and 0.031° (~3.44 km) per year for median latitude of reported cases. *Per contra*, *V. alginolyticus* and *V. parahaemolyticus* showed the largest distributional changes, with shifts of 0.633° (~70.3 km) and 0.550° (~61.1 km) per year to the latitude of their northernmost reported cases and 0.178° (~19.8 km) and 0.447° (~49.6 km) per year to the latitude of their median reported cases, respectively. Therefore, while the changes between climate and vibriosis coincide, not all *Vibrio spp*. were affected equally.

As vibriosis cases continue to rise in northern latitudes of the eastern U.S., there is growing concern about the potential larger outbreaks, particularly in densely populated coastal states such as New York and New Jersey, which rank among the top five in coastal population (71), as well as other northern states. The potential risk of vibriosis becoming endemic in these areas is therefore significant. While the findings of this study are specific to the U.S. eastern and southern coasts, increasing vibriosis incidence has been documented globally, including Japan and Southeast Asia (72), northern Europe (73), Peru and Alaska (74), and Malaysia (75). Although the models developed here may not directly transfer to other regions, the modeling framework and environmental data sources are broadly applicable and can support global efforts to predict the risk of vibriosis and other waterborne diseases.

Results of this study show a strong connection between environmental conditions and reported vibriosis cases. Specifically, the XGBoost models identified vibriosis accurately for all *Vibrio spp*. (average accuracy >60%) using only environmental data as model input, especially important given the large spatial extent of the data and large class imbalance. Models for *V. vulnificus* and *V. alginolyticus* performed best, likely due to the fact that vibriosis caused by the two species was almost all nonfoodborne infections, compared to *V. parahaemolyticus*, which is generally associated with foodborne infection. Nonfoodborne infections perhaps may be more easily incorporated into predictive algorithms, since environmental conditions are strongly indicative of environmental conditions where the infection occurred. Foodborne infections, in contrast, involve greater variability, since contamination of seafood can occur during transport and improper storage can result in bacterial contamination.

The importance of SST and SSS as indicators for vibriosis is evident, given that they are the two most important variables in each model and relatively high SHAP values compared to other variables. These variables should be central to any future models, particularly given the observation that these parameters along the coastline and coastal vibriosis cases have both increased significantly and concurrently. While precipitation and phytoplankton had highly complex nonlinear relationships with vibriosis, they did show high relative importance and high SHAP values, indicating that while the relationship is complex, they are important to include in future models. It is useful to include them in models to differentiate *Vibrio sp*., since most of the differences between models were captured by response to phytoplankton size and precipitation.

Other investigators have reported an increase in *Vibrio spp*. proliferation related to increasing temperature (22). Ocean warming has contributed to a 0.93°C (0.73°C–1.04°C) increase in ocean temperatures in 2013–2022 compared to 1850–1900 (76). Here, we observed an increase of annual mean SST within 20 km of the U.S. eastern and southern coasts of *ca.* 1.33°C (1990–1996 compared to 2015–2019), with increases greatest in summer months (between *ca*. 2.47°C and 2.67°C; see supplemental material). With climate change linked to both of these increased temperatures and SST, the strongest indicator of vibriosis in XGBoost models, it is concluded that ocean warming is a very strong contributor to the observed change in vibriosis case distribution over time.

Salinity is important with respect to vibriosis modeling and clustering. Previous studies reported *Vibrio spp*. have a preferred salinity range, and for some species, the range is highly specific. *V. cholerae*, for example, grows optimally at salinities between 15 and 25 ppt (77), while for *V. alginolyticus*, optimal growth is achieved at salinity concentrations between 30 and 35 ppt (78). *V. vulnificus* and *V. parahaemolyticus* isolates from the Chesapeake Bay grow best at 10–15 ppt salinity (13) and *V. mimicus* at 4 ppt in some aquatic environments, e.g., in Dhaka, Bangladesh, and Okayama, Japan (79). Thus, the salinity results of this study align well with previous reports, except *V. mimicus*, for which limited data are available with respect to salinity tolerance. Changes in ocean salinity since the mid-20th century have been documented (76) and an increasing frequency of salinity variability predicted (68).

A growing body of evidence has clarified the role of environmental parameters in shaping the occurrence and distribution of *Vibrio spp*., notably *V. cholerae*, *V. parahaemolyticus*, *V. vulnificus*, and *V. alginolyticus*, all of which are naturally occurring in aquatic ecosystems (2). These bacteria typically thrive in warm, moderately saline waters. However, some *Vibrio spp*., notably *V. parahaemolyticus* and *V. alginolyticus*, exhibit broad salinity tolerance, and others exist in hypersaline waters (78, 80, 81). In the study reported here, an increased salinity was noted along the coastline, pushing these regions above an optimal range for the proliferation of *Vibrio spp*. However, in estuarine and brackish environments, where the baseline salinity is lower, such increases may create more favorable conditions for the growth of *Vibrio spp*. The case reports served as useful proxies for environmental proliferation, and a more pronounced shift in vibriosis distribution in the dynamic coastal regions can be expected.

The contributions of chl-a and phytoplankton to *Vibrio spp*. growth and proliferation are complex, as determined by the models, with each *Vibrio sp*. having a specific optimal chl-a relationship with respect to its distribution in the environment. The k-means clustering analysis showed two clusters of chl-a concentration, *ca*. 10–20 mg/L with differing salinities for the clusters. However, clusters of cases associated with chl-a concentrations of *ca*. 4–7 mg/L were noted. Clearly, increased nutrient and phytoplankton concentrations have been shown to be associated with increased abundance of *Vibrio spp*. (21, 30, 82, 83). Nonetheless, the results of the study reported here indicate that the relationship is complex, with multiple factors involved in *Vibrio spp*. replication and abundance in the environment. For example, the microphytoplankton is composed of diatoms and dinoflagellates, the picophytoplankton pico-eukaryotes, prokaryotes, and *Prochlorococcus spp*., and the nanophytoplankton green algae and prymnesiophytes (haptophytes) (63). Understanding their thresholds indicative of vibriosis potential for each *Vibrio spp*. in future studies will provide insight into the complexity of communities with which *Vibrio spp*. are associated.

### Study limitations

Primary limitations of this study derive from the uncertainty of the exact location of case initiation and the proximity of cases to given environmental conditions. That is, cases are reported correctly in the counties of interest, but there is the possibility of lagged reporting of cases, unreported cases, or cases having no association with source environmental conditions, i.e., imported seafood or hospital visits not in the county where the case occurred. Such factors have the potential to introduce spatial and temporal uncertainty, which can reduce the precision of the association between environmental parameter and case occurrence. In particular, foodborne infections linked to transported seafood will obscure the local environmental driver, while nonfoodborne cases (predominantly wound infections) are more likely to reflect exposure at or near the site of reporting.

Additionally, the application of the models is limited by training models using random under-sampling on imbalanced data sets. Applying them to real-world scenarios, where outcomes are highly imbalanced, would provide lower precision and recall than reported here. This methodological tradeoff improves model interpretability but reduces performance when applied to unbalanced population-level data.

However, the results indicate reliable use of environmental parameters to differentiate case presence and absence. Importantly, model outputs should be interpreted as identifying ecological suitability for vibriosis, rather than predicting exact case counts. Future efforts should be able to incorporate improved case attribution, higher-resolution exposure data, and alternative approaches for handling class imbalance to enhance predictive accuracy and generalizability on a global scale.

### Conclusions

Incidence of vibriosis has increased over the past 30 years and has also expanded with respect to geographic distribution, notably along the U.S. eastern coast. The northernmost reported case latitude increase for *V. parahaemolyticus* (61.0 km/year), *V. vulnificus* (39.3 km/year), *V. alginolyticus* (70.3 km/year), *V. cholerae* non-O1, non-O139 (54.6 km/year), *V. fluvialis* (60.2 km/year), and *V. mimicus* (42.1 km/year) cases is significant. The total number of cases of vibriosis attributed to these six species is *ca*. 500 annually. This study provides a foundation for assessing environmental drivers of vibriosis associated with climate change, notably SST, SSS, and chl-a. Predictive risk models for non-cholera *Vibrio* spp. infections and those caused by less commonly reported species, such as *V. alginolyticus*, *V. fluvialis*, and *V. mimicus*, can now be constructed. In summary, modeling efforts will be critical in addressing future changes in the incidence of vibriosis.

## SUPPLEMENTAL MATERIAL

Supplemental figures aem.00716-25-s0001.docx Figures S1 to S12.

ASM does not own the copyrights to Supplemental Material that may be linked to, or accessed through, an article. The authors have granted ASM a non-exclusive, world-wide license to publish the Supplemental Material files. Please contact the corresponding author directly for reuse.
