# Supplementary material for: Machine learning in understanding environmental variability of vibriosis in coastal waters
Source: Appl Environ Microbiol. 2025 Aug 14;91(9):e00716-25. doi: 10.1128/aem.00716-25 (PMC12442386; doi:10.1128/aem.00716-25)
Supplement: Supplemental figures — Figures S1 to S12. [file aem.00716-25-s0001.docx]

**Supplemental Materials**


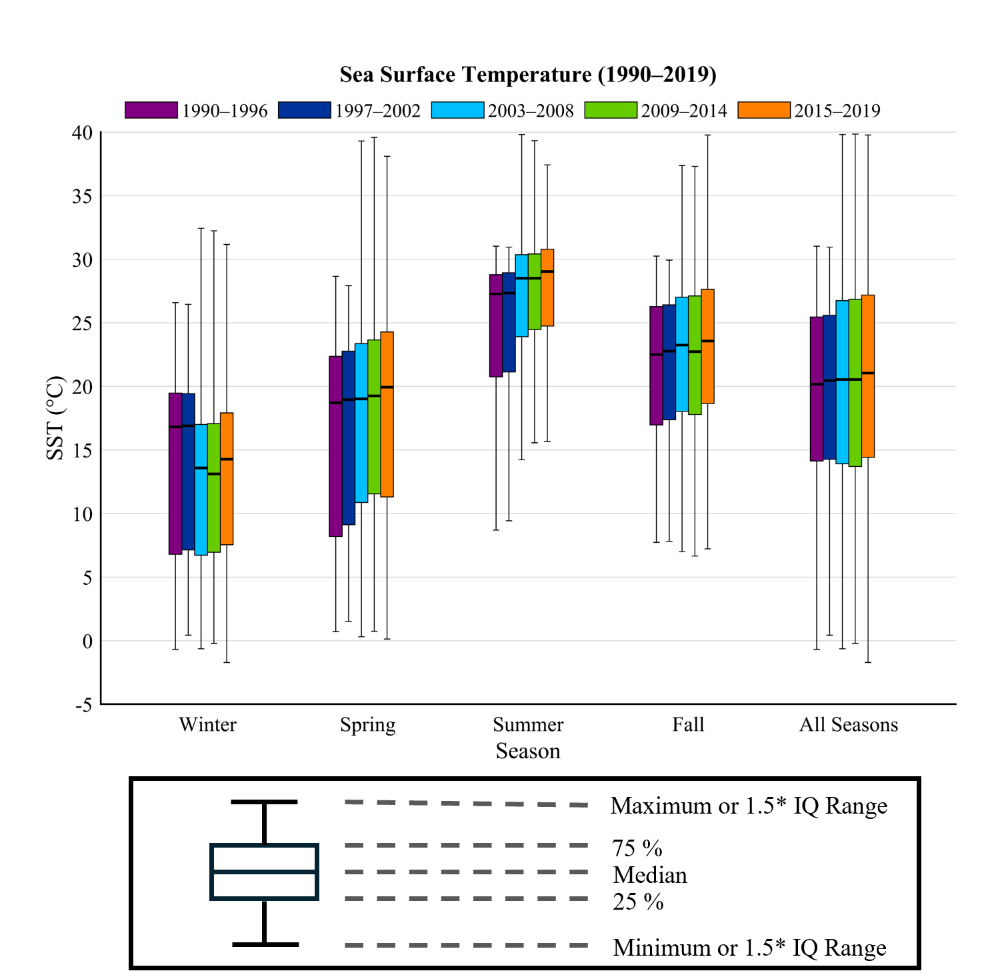


**Supplemental Figure 1.** Box and whisker plots of SST along the US eastern and southern seaboard, 1990-2019.


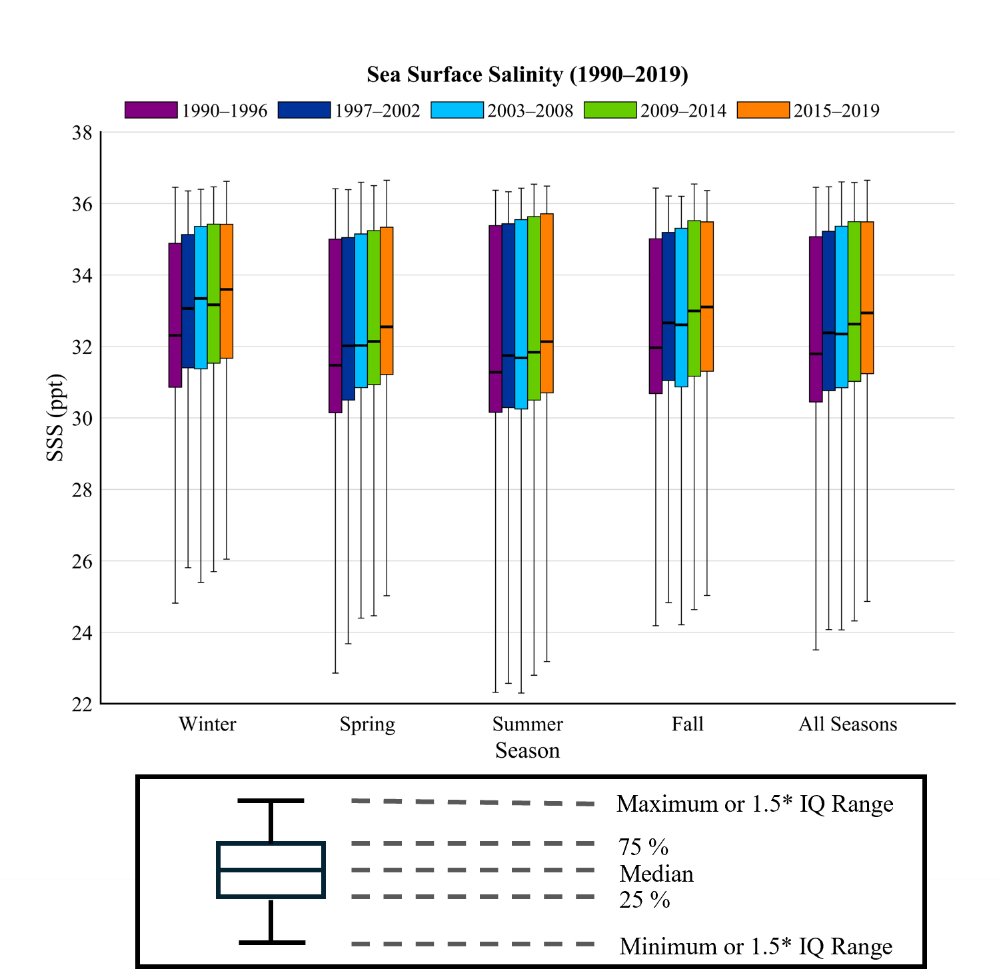


**Supplemental Figure 2.** Box and whisker plots of SSS along the US eastern and southern seaboard, 1990-2019.


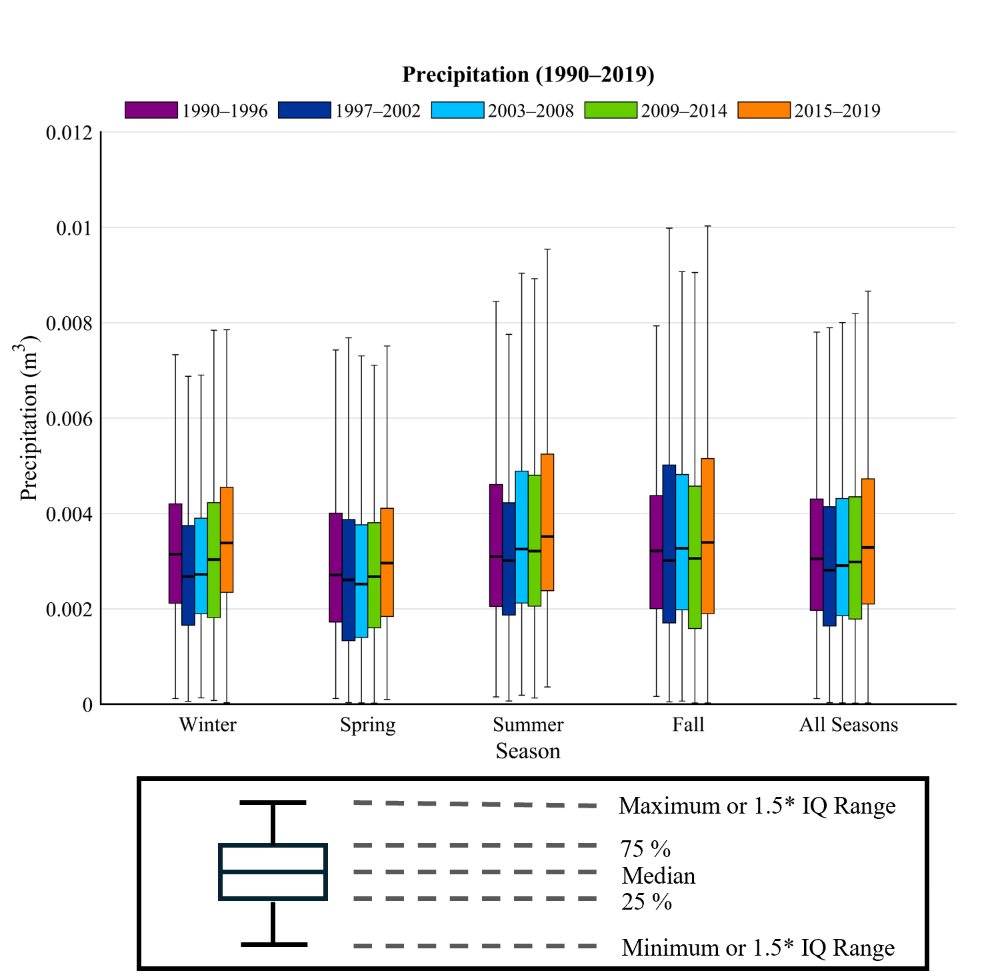


**Supplemental Figure 3.** Box and whisker plots of precipitation along the US eastern and southern seaboard, 1990-2019.


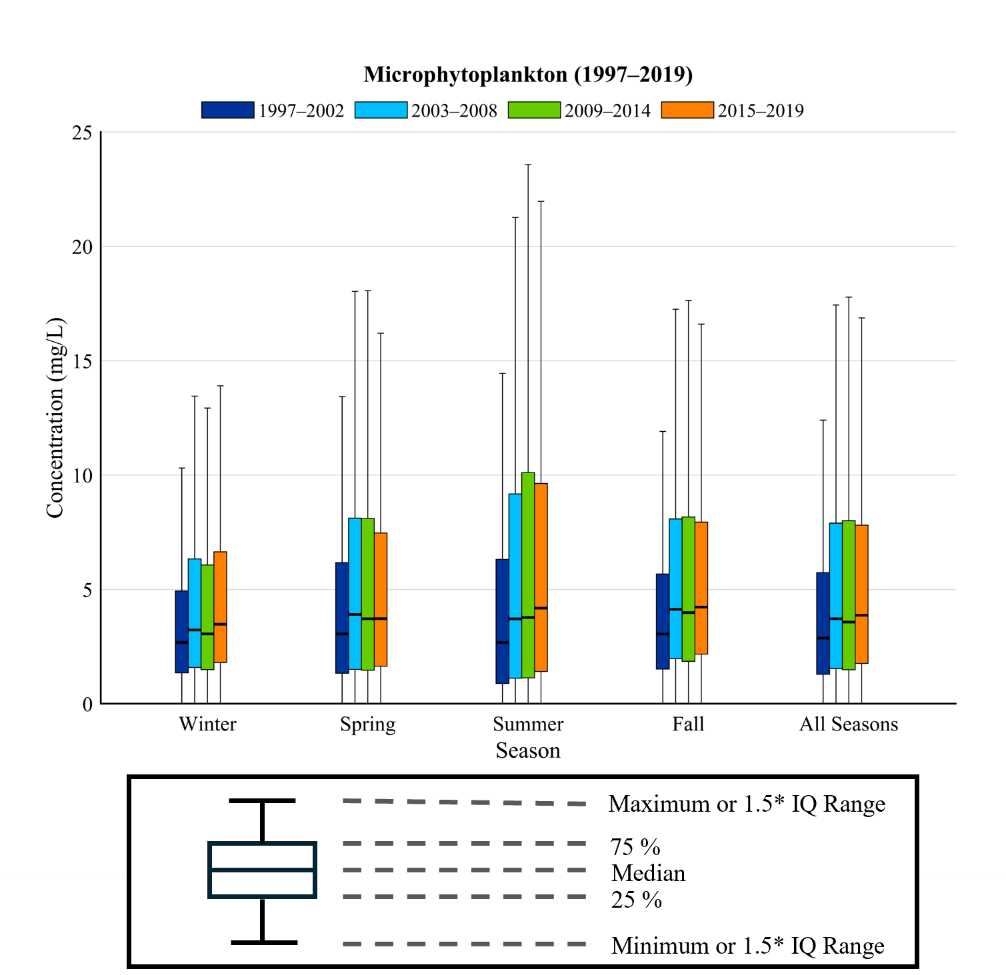


**Supplemental Figure 4.** Box and whisker plots of microphytoplankton along the US eastern and southern seaboard, 1990-2019.


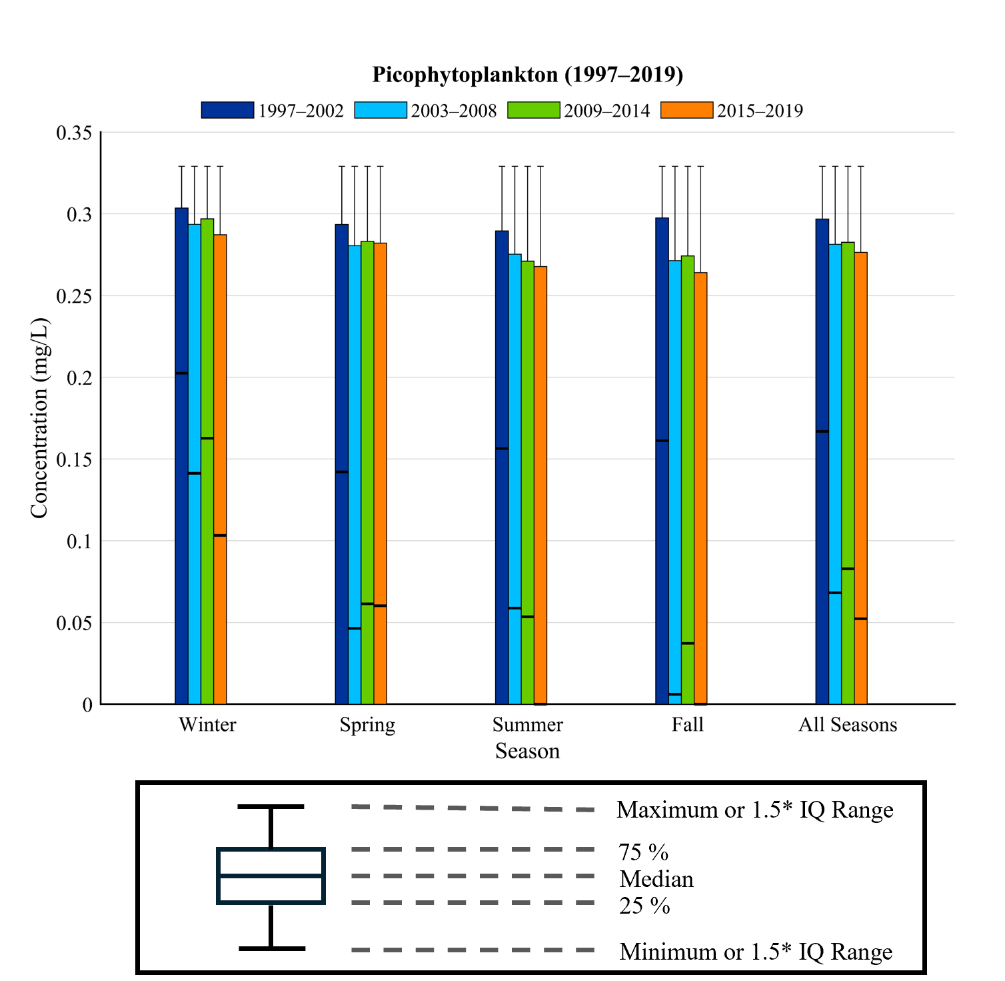


**Supplemental Figure 5.** Box and whisker plots of picophytoplankton along the US eastern and southern seaboard, 1990-2019.


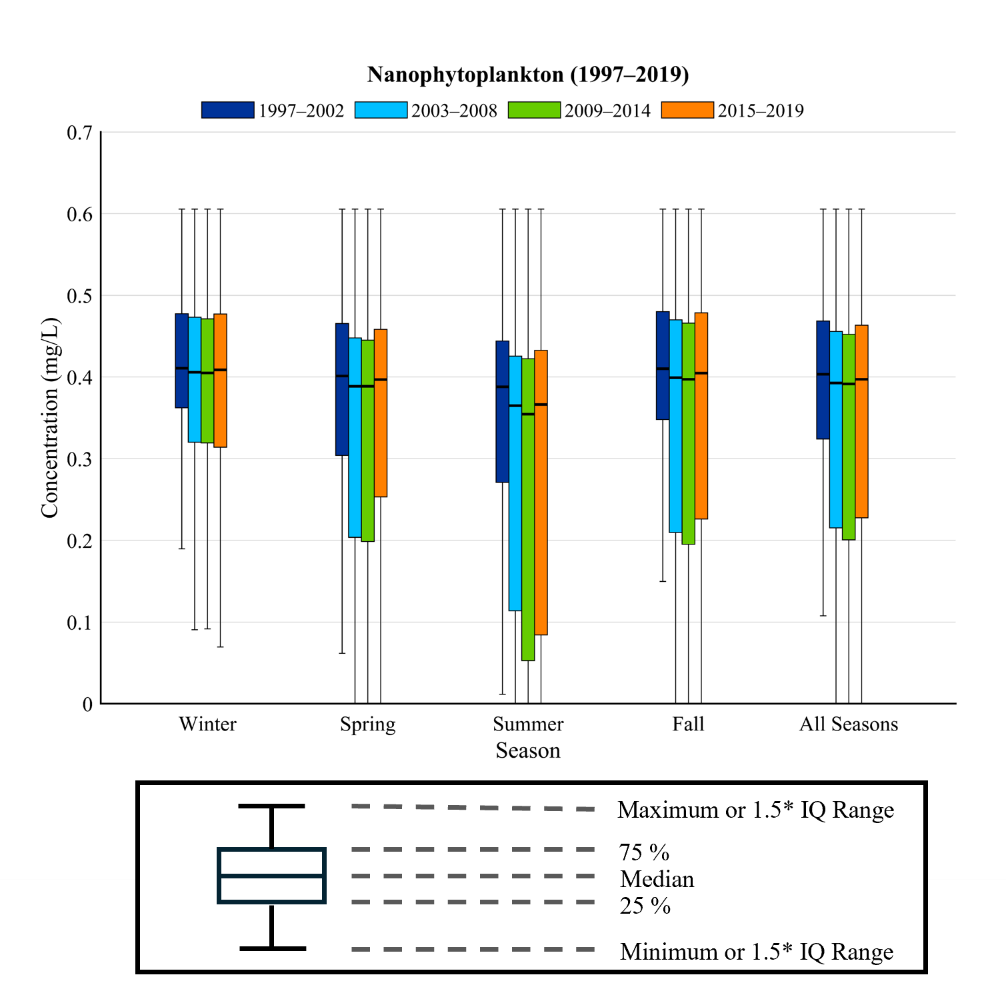


**Supplemental Figure 6.** Box and whisker plots of nanophytoplankton along the US eastern and southern seaboard, 1990-2019.


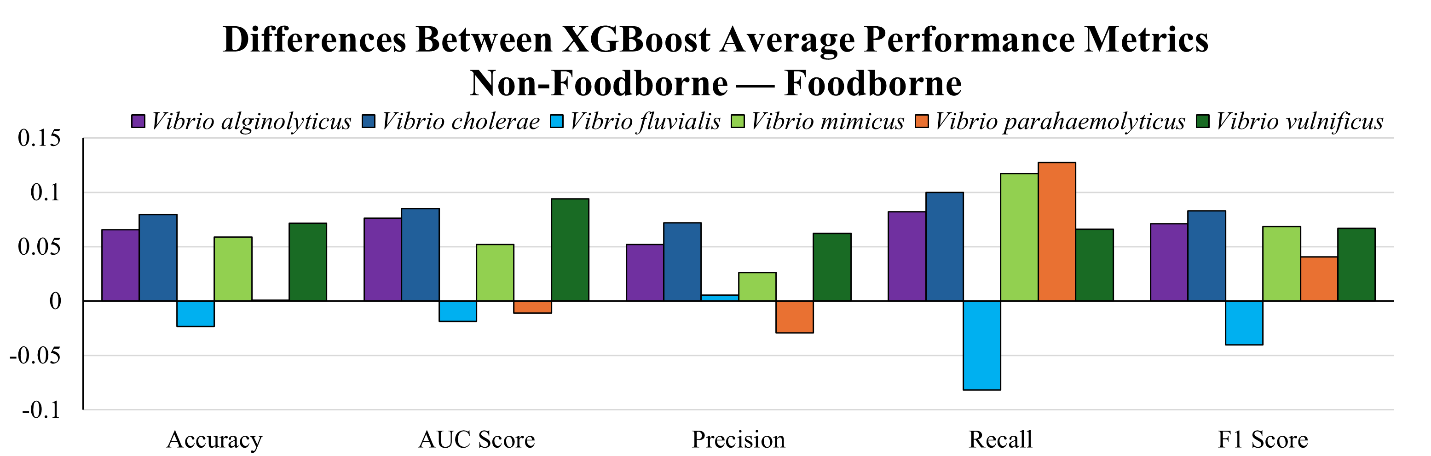


**Supplemental Figure 7.** Differences between model performance metrics using only nonfoodborne cases and only foodborne cases.


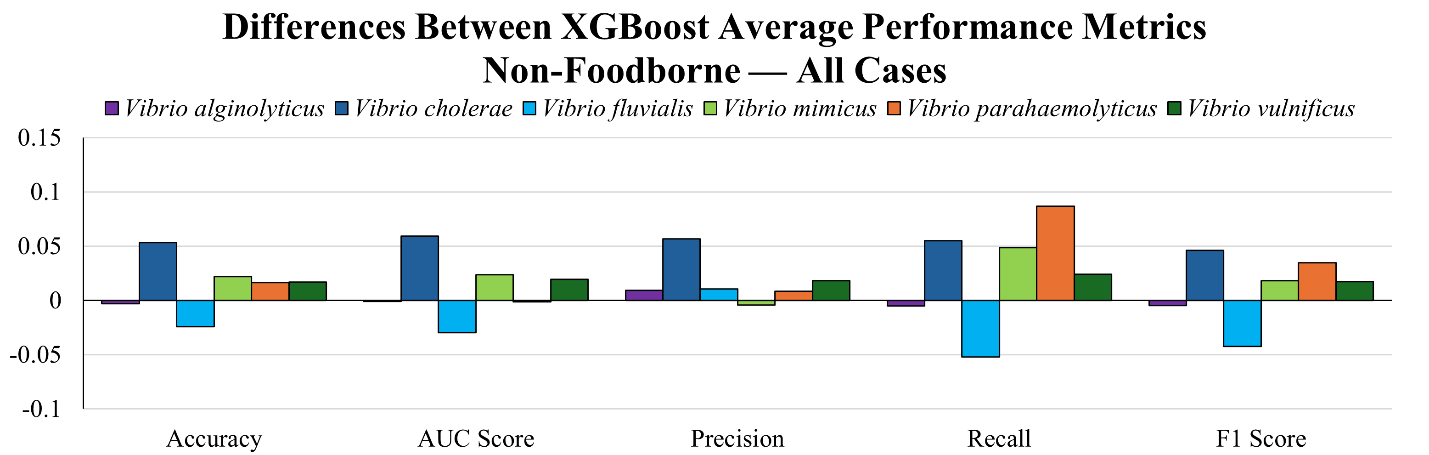


**Supplemental Figure 8.** Differences between model performance metrics using only nonfoodborne cases and all cases.


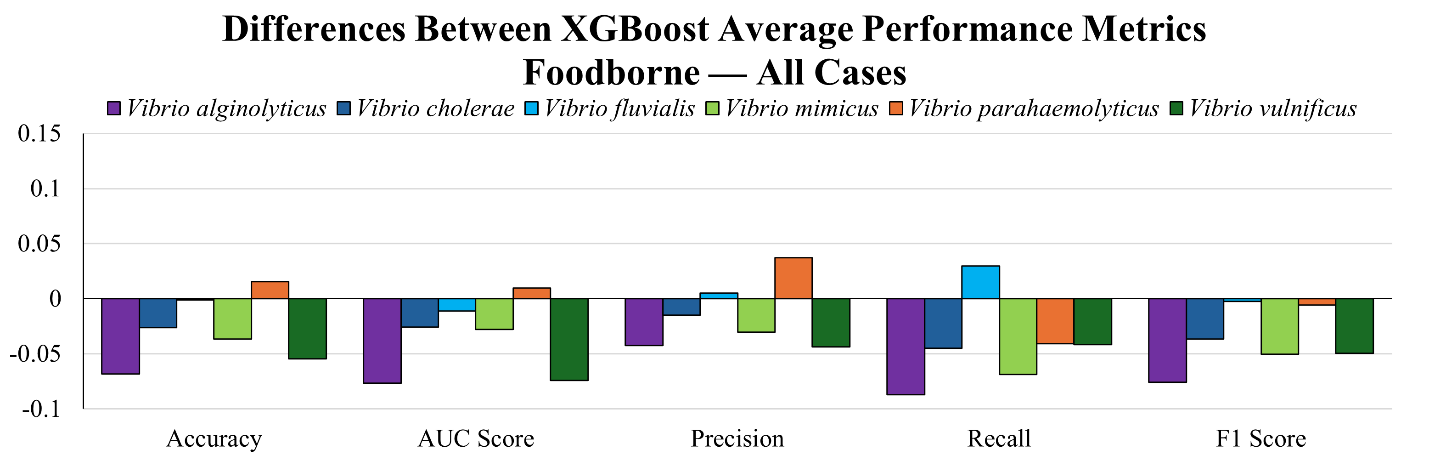


**Supplemental Figure 9.** Differences between model performance metrics using only foodborne cases and all cases.


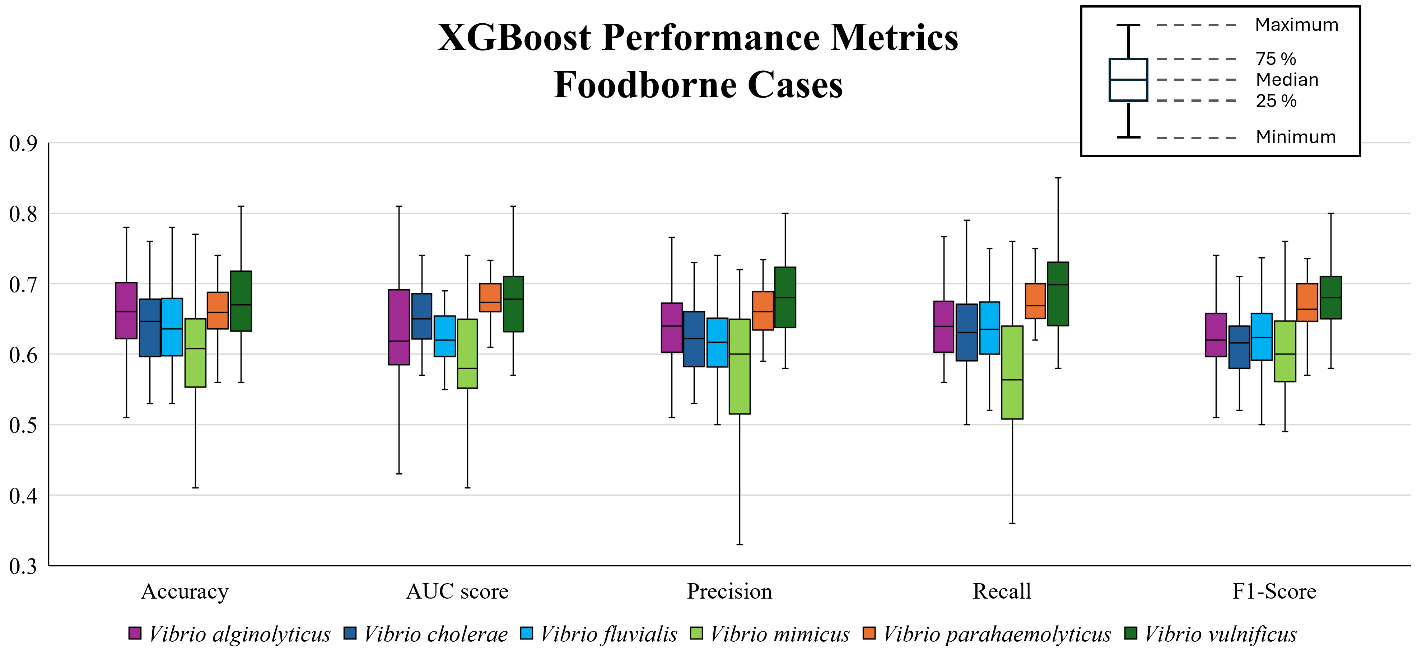


**Supplemental Figure 10.** Box and whisker plot of model performance metrics using only foodborne cases.


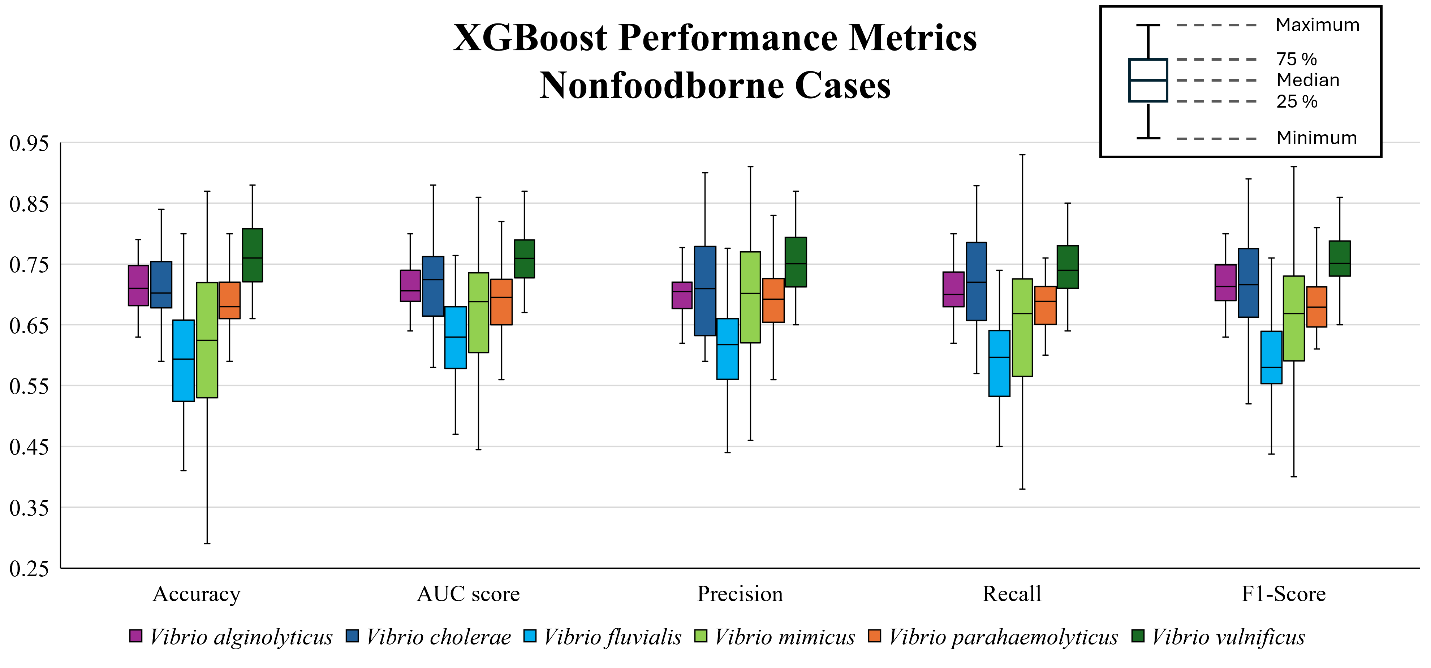


**Supplemental Figure 11.** Box and whisker plot of model performance metrics using only nonfoodborne cases.


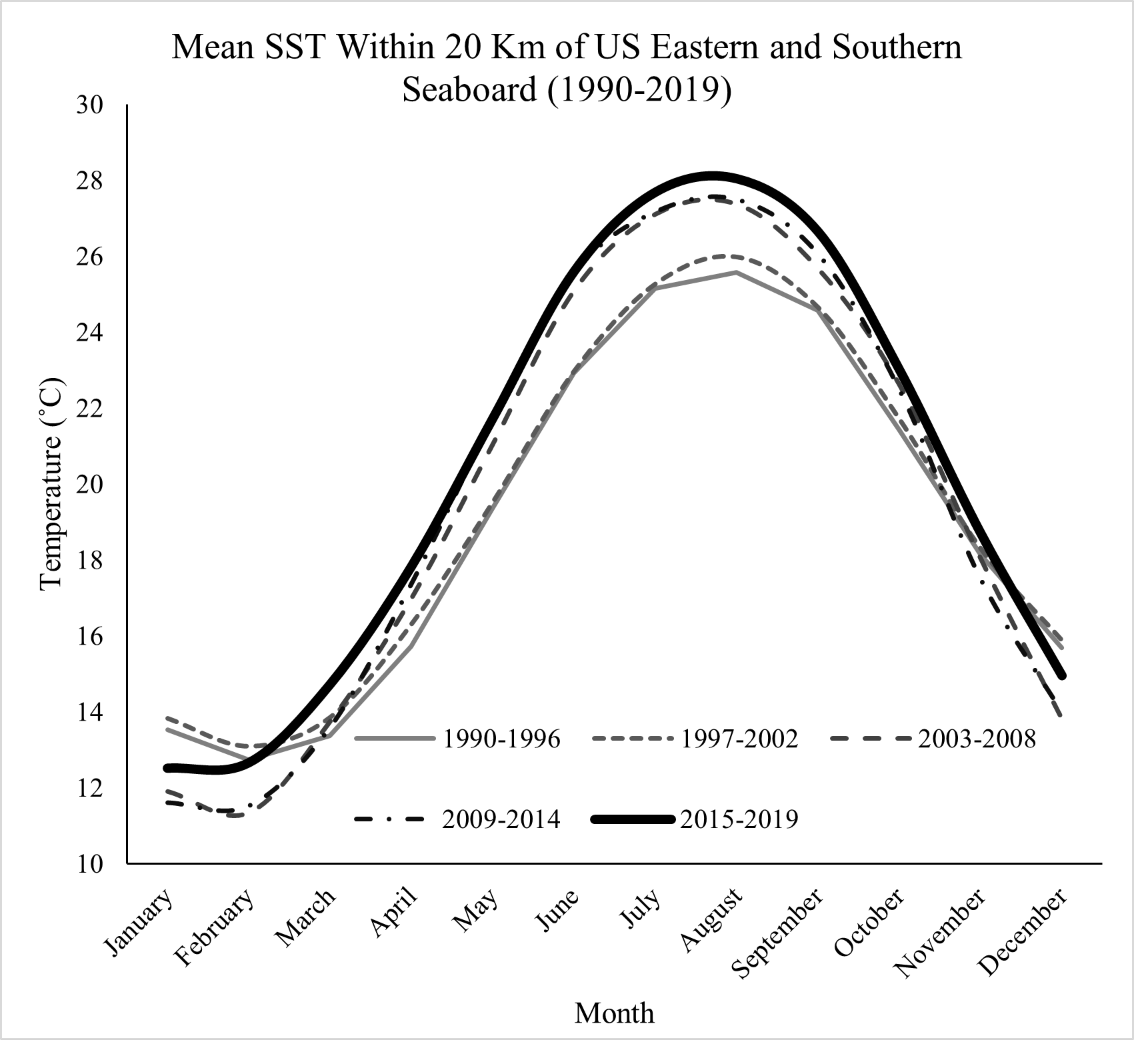


**Supplemental Figure 12.** Mean SST within 20 km of the US eastern and southern seaboard, 1990-2019.
